# A duck RH panel and its potential for assisting NGS genome assembly

**DOI:** 10.1186/1471-2164-13-513

**Published:** 2012-09-28

**Authors:** Man Rao, Mireille Morisson, Thomas Faraut, Suzanne Bardes, Katia Fève, Emmanuelle Labarthe, Valérie Fillon, Yinhua Huang, Ning Li, Alain Vignal

**Affiliations:** 1 UMR INRA/ENVT Laboratoire de Génétique Cellulaire, INRA, Castanet-Tolosan, 31326, France; 2 State key laboratory for agro-biotechnology, China Agricultural University, Beijing, 100193, People's Republic of China

**Keywords:** RH mapping, NGS, Sequencing, Duck, Scaffold, Assembly

## Abstract

**Background:**

Owing to the low cost of the high throughput Next Generation Sequencing (NGS) technology, more and more species have been and will be sequenced. However, *de novo* assemblies of large eukaryotic genomes thus produced are composed of a large number of contigs and scaffolds of medium to small size, having no chromosomal assignment. Radiation hybrid (RH) mapping is a powerful tool for building whole genome maps and has been used for several animal species, to help assign sequence scaffolds to chromosomes and determining their order.

**Results:**

We report here a duck whole genome RH panel obtained by fusing female duck embryonic fibroblasts irradiated at a dose of 6,000 rads, with HPRT-deficient Wg3hCl_2_ hamster cells. The ninety best hybrids, having an average retention of 23.6% of the duck genome, were selected for the final panel. To allow the genotyping of large numbers of markers, as required for whole genome mapping, without having to cultivate the hybrid clones on a large scale, three different methods involving Whole Genome Amplification (WGA) and/or scaling down PCR volumes by using the Fluidigm BioMark^TM^ Integrated Fluidic Circuits (IFC) Dynamic Array^TM^ for genotyping were tested. RH maps of APL12 and APL22 were built, allowing the detection of intrachromosomal rearrangements when compared to chicken. Finally, the panel proved useful for checking the assembly of sequence scaffolds and for mapping EST located on one of the smallest microchromosomes.

**Conclusion:**

The Fluidigm BioMark^TM^ Integrated Fluidic Circuits (IFC) Dynamic Array^TM^ genotyping by quantitative *PCR* provides a rapid and cost-effective method for building RH linkage groups. Although the vast majority of genotyped markers exhibited a picture coherent with their associated scaffolds, a few of them were discordant, pinpointing potential assembly errors. Comparative mapping with chicken chromosomes GGA21 and GGA11 allowed the detection of the first chromosome rearrangements on microchromosomes between duck and chicken. As in chicken, the smallest duck microchromosomes appear missing in the assembly and more EST data will be needed for mapping them. Altogether, this underlines the added value of RH mapping to improve genome assemblies.

## Background

The development and commercialization of next-generation massively parallel DNA sequencing approaches, by dramatically decreasing the cost of sequencing, have revolutionized genomic research. The main innovation of NGS, as compared to Sanger sequencing, is the parallelisation of the process, allowing between a few thousands and up to millions of sequencing reactions to be processed simultaneously. The three main NGS technologies available on the market use different approaches for library construction, template immobilisation and sequencing reaction, but the basic principles remain the same. NGS approaches also have some drawbacks compared with Sanger sequencing: (1) sequence reads produced currently by NGS (100 bp for Illumina, 500 bp for 454) are shorter than Sanger sequencing reads (1000 bp) and have a higher error rate, making the sequence assembly more problematic; (2) the pairing of reads in Sanger sequencing is limited by the size of the DNA fragments that can be inserted in cloning vectors, ranging from 1–2 kb or less up to 100–200 kb (plasmids, fosmids, BAC), whereas pairing of reads is limited to 40 kb with NGS, limiting the average assembled scaffold length and leading to more difficulty in segmental duplication and copy number variation detection; (3) as a consequence, a higher sequencing depth is required for assembly and a very high number of small scaffolds are produced. Nevertheless, the sequencing and *de novo* assembly of a Chinese individual [1] proved the feasibility of sequencing and assembling whole genomes by NGS. Many species have been sequenced and/or resequenced by NGS, such as the giant panda *Ailuropoda melanoleura*[2], the silk worm *Bombyx mori*[3], the cucumber *Cucumis sativus*[4], the chicken *Gallus gallus domesticus*[5,6], the turkey *Meleagris gallopavo*[7] and the Mallard duck *Anas platyrhybchos domesticus* (Huang *et al.*, in prep).

The Pekin duck (*Anas platyrhynchos*, APL) is an obvious target for detailed genomic studies due to its agricultural importance [8-10] as well as for its role as a natural reservoir of all influenza A viruses. It can usually carry the infection with no sign of disease and thus propagates the virus to other bird species and potentially to mammals such as pigs or humans [11-15]. The duck genome presents most of the characteristics encountered in birds, which are: (i) a more compact genome, one third the size of a typical mammalian one, (ii) a large number of chromosomes (2n = 80), (iii) the presence of macrochromosomes and microchromosomes, the latter being as small as a few Mb [16] and (iv) the females are the heterogametic sex (ZW) and males the homogametic one (ZZ). Due to its importance both in the economic and scientific fields, the sequencing of the Pekin duck genome was initiated in 2008 using the same strategy recently published for the giant panda [2] at the Beijing Genomics Institute (BGI). The sequence reads provided a depth of 65X and a total of 78,487 scaffolds were assembled in which the N50 scaffold was 1.2 Mb and the largest was 5.9 Mb in length (Huang *et al.*, in prep). However, owing to the lack of a clone-based physical map and other supplementary mapping data, apart from a first generation genetic map composed of 155 microsatellite markers, 115 of which located in only 19 linkage groups spanning 1353.3 cM [17], it is possible to assign only very few assembled scaffolds to chromosomes.

Several studies have shown that birds seem to have a slower rate of chromosome rearrangements than mammals, with only very little inter-chromosomal rearrangements [18-23]. Between chicken and zebra finch, whole genome comparisons revealed 114 tentative intrachromosomal rearrangements (56 inversions and 58 translocations) in which some were confirmed by FISH (Fluorescent *In Situ* Hybridization) experiments [24,25]. Recently, Zhang *et al.* (2011) provided confirmed evidence for 20–27 major rearrangements between turkey and chicken, almost all of which are inversions [26]. The mean reported phylogenetic distance between chicken and turkey is 47 million years, whereas it is 81 between chicken and duck [27], so the number or rearrangements reported between chicken and turkey provide the minimum level of difference expected between chicken and duck. To date, only one interchromosomal difference has been reported between the chicken and the duck karyotypes, with APL4 and APL10 corresponding to GGA4q and GGA4p respectively [21]. This interchromosomal rearrangement explains the difference in diploid chromosomal number between the two species, which is 2n = 78 in chicken and 2n = 80 in duck and therefore the nomenclature for numbering the duck chromosomes follows mainly that of chicken. Macrochromosomes APL1 to APL9 correspond to GGA1 to GGA9, then APL10 corresponds to GGA4p and finally, the rest of the karyotype is offset by one, with GGA10 corresponding to APL11 and so on. Cross-species fluorescent in-situ hybridization (FISH) studies using chicken BAC clones on duck metaphase spreads showed only a few large scale intrachromosomal rearrangements concerning the largest chromosomes [23,28]. All this demonstrates a high karyotype stability despite 80 million years of divergence between the two species [29,30].

As a first attempt to order the duck sequence scaffolds, we aligned the 7,205 ones larger than 1 kb to the current chicken assembly using the Narcisse database [31,32] and successfully positioned 1,787 of them. This still leaves a large number of scaffolds to assign and also means that the ordering of the duck scaffolds and genes we obtained will follow the chicken genome and will be wrong whenever large- or small-scale rearrangements will have happened between the two species.

To assemble the scaffolds in an order corresponding to the real duck chromosomes, several approaches can be used. High density SNP genetic maps allow high precision in mapping. However, the SNP markers need first to be discovered by a sequencing approach, such as published by Kraus *et al.* (2011) [33] and must be informative in a reference population to be used for mapping. However, despite several thousand SNP discovered to date [33], only a small subset of 384 were genotyped [34], mainly due to the high cost that would have been required for additional markers. Finally, out of these, only a small subset was informative in mapping populations (R. Kraus, R. Crooijmans, personal communication), which will allow only for low resolution maps and poor marker ordering. Furthermore, for high precision mapping, very large populations counting several hundred individuals are required, yet again increasing cost and labour. Further sequencing for SNP detection and a consortium for generating a SNP chip would help improve genetic maps in duck and may happen in the future. Physical maps can be based on the mapping of BAC clones by FISH for chromosome assignment and large-scale ordering. The BAC clones from large libraries can be used for contig construction by fingerprinting or high throughput hybridization. End-sequencing of the clones allow linking sequence scaffolds together. BAC contig maps are thus usually a backbone to the sequence assembly. A BAC library has been made for Duck [35], but to the best of our knowledge, there are no plans yet to build physical maps. In this context, radiation hybrid mapping can be an excellent complementary mapping approach, as it does not require complex marker development and large-scale genotyping. Any STS can be placed on the map by simple PCR on as little as 90 hybrids. Thus with a minimal effort, maps with a resolution intermediate between the genetic and the BAC contig maps can be constructed to propose a correct chromosomal assignment and ordering of scaffolds. We report here the production of a duck whole genome radiation hybrid panel and demonstrate its utility to verify the quality of sequence scaffolds and for assigning and positioning scaffolds onto chromosomes. Large-scale culture of radiation hybrid clones is a time-consuming process and moreover causes the loss of donor fragments in the hybrids. To avoid the necessity of cultivating the radiation hybrid clones at a large scale, we tested three approaches. One involves whole genome amplification (WGA) and conventional genotyping by PCR and gel electrophoresis and the other two use minute amounts of DNA and Fluidigm BioMark^TM^ IFC Dynamic Array^TM^ genotyping by quantitative *PCR*. The advantage of the Fluidigm approaches is low cost combined with simple and rapid high-scale genotyping.

## Results

### Generation and characterisation of a duck radiation hybrid panel

Duck radiation hybrids were obtained by fusing female duck embryonic fibroblasts irradiated at a dose of 6,000 rads, with HPRT-deficient hamster cells from the Wg3hCl_2_ cell line. Five fusion experiments were carried out to produce 225 duck radiation hybrids, suggesting that one hybrid clone was recovered per 289,000 duck fibroblasts, corresponding to an average fusion efficiency of 3.46 x 10^-6^ clone per duck fibroblast. Retention frequencies in the hybrids were estimated by using a set of 31 microsatellite markers distributed along the duck genome, whose positions were estimated on the basis of a low resolution genetic map (Marie-Etancelin *et al.*, in prep). Genotyping was performed by conventional PCR followed by agarose gel electrophoresis. As the ancestral chromosomes 4 and 10, fused in chicken to give GGA4q and GGA4p respectively, remain separated in duck as APL4 and APL10 [23,28], care was taken to choose one marker located on APL10 and 3 others on APL4. As microchromosomes and the regions close to centromeres were reported to be better retained in chicken radiation hybrids [36-38], we decided to focus more on macrochromosomes and use a higher proportion of markers from macrochromosomes. Altogether, 20 markers from macrochromosomes 1 to 7 and chromosome Z were selected and the rest (11 markers) were from identified microchromosomes. By using the genetic maps and comparative mapping with chicken, we avoided the clustering of markers.

As a result from genotyping, we estimated the average retention frequency of duck genome fragments in the 225 hybrids to be 15.3% for the whole genome, with unequal values for macrochromosomes (10.2%) and microchromosomes (21.8%). Previous estimations showed that a panel of 100 hybrids with marker retention frequencies between 20 and 50% are sufficient to build maps of chromosomes at a reasonable resolution [39]. Almost 50% of our duck-hamster hybrids have an average retention frequency over 15%, being thus potential candidates for the final panel. Finally, the 90 hybrids selected for the definitive panel were chosen with the highest possible marker retentions for macrochromosomes while maintaining good values for microchromosomes. Final retention frequency values are 23.6% genome-wide, with specific values of 20.2% for the macrochromosomes and 28.1% for the microchromosomes.

### Testing three different RH strategies for mapping macrochromosomes and medium size microchromosomes

Several thousand markers are needed to build genome-wide maps, requiring large amounts of DNA, usually prepared by large-scale culture of the radiation hybrid clones. However, this is a time-consuming task and moreover, donor chromosome fragments are lost during the culture process. To avoid this, we tested three alternative methods allowing minute amounts of DNA from the hybrids to be used. These were based either on whole genome amplification (WGA) by Multiple Displacement Amplification (MDA) of the DNA from the hybrid clones and/or on scaling down the PCR to 7 nl, allowing the DNA requirements to be as little as 70 pg, by using the Fluidigm BioMark^TM^ IFC Dynamic Array^TM^ genotyping by quantitative PCR (FLDM) [40]. The three conditions were thus: (i) WGA-PCR, in which the DNA from the hybrid clones was amplified by WGA and the genotyping performed by conventional PCR followed by agarose gel electrophoresis; (ii) WGA-FLDMqPCR, in which the WGA-amplified DNA was used for genotyping by quantitative real-time PCR in 7 nl reaction volumes and (iii) Pre-ampFLDMqPCR, in which the DNA from the clones was used directly without WGA, which was replaced by a more specific pre-amplification step using the 96 primer pairs for the 96 loci studied in one Fluidigm BioMark^TM^ run (see methods).

**Figure 1:**
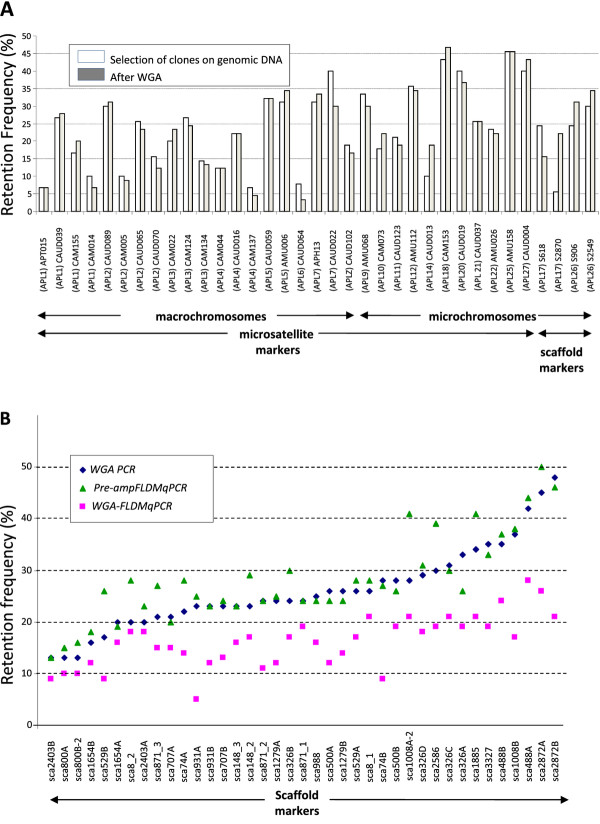
**Estimations of duck genome retention in the RH clones. A:** retention frequencies of thirty-one microsatellite markers and four scaffold markers before (white) and after (grey) whole genome amplification. The test was done on the 90 selected hybrids by conventional Agarose genotyping. The expected chromosome locations of the markers (given in brackets) are derived from the chicken/duck comparative FISH mapping and a duck genetic map (Marie-Etancelin *et al.,* in prep) for the microsatellite markers and according to comparative genomic data given by the Narcisse software [32] for the scaffold markers. **B**: Retention frequencies of thirty-nine scaffolds markers obtained using three different genotyping strategies. The thirty-nine scaffold markers were genotyped using either (i) the amplified panel with conventional agarose genotyping (blue: WGA-PCR), (ii) the non amplified panel and genotyping with the Fluidigm BioMark gene expression dynamic array (green: Pre-ampFLDMqPCR) or (iii) the amplified panel and genotyping with the Fluidigm BioMark^TM^ IFC Dynamic Array^TM^ genotyping by quantitative PCR without any pre-amplification step (purple: WGA-FLDMqPCR). The markers are distributed along the X axis from the lowest to the highest retention frequencies obtained by the first method (the amplified panel with conventional agarose genotyping WGA-PCR in blue).

Whole genome amplifications with MDA were performed for all the 90 selected radiation hybrids. Each sample was amplified in three replicates which were pooled together to avoid representation bias in the final working panel. We obtained a 1000 X amplification efficiency, with more than 150 μg of WGA-DNA obtained for each hybrid, from 150 ng of starting DNA. As the smallest microchromosomes have always proved difficult to sequence and to clone in chicken, we supposed a bias could also exist for WGA. To check for correct amplification of microchromosomes, we designed markers from two scaffolds located on APL17, orthologous to GGA16 and containing the two major histocompatibility complex (MHC) gene clusters and the Nucleolar Organizing Region (NOR) rRNA genes, and from two scaffolds located on APL26, according to comparative genomic data given by the Narcisse database [31]. These 4 markers were added to our first set of 31 microsatellite markers we used primarily for selecting the 90 clones for the panel. Genotyping this set of markers on the WGA-DNA of the 90 hybrids demonstrated average retention frequencies of 23.8% for the whole genome, with 19.3% for the macrochromosomes and 29.9% for the microchromosomes. On average, retention frequencies are very close to those observed before the WGA (Figure 1A). However, retention frequency of S2870 located on APL17, S906 and S2549 located on APL26 were increased after WGA, especially for S2870. In contrast, a slight retention loss was found for S618, the other scaffold marker from APL17. Thus amplifying the panel by WGA and genotyping by the conventional PCR and agarose gel electrophoresis approach (WGA-PCR) appears to be a good option for mapping without having to perform large-scale culture of the hybrids, at least for macrochromosomes and medium-sized microchromosomes.

**Figure 2:**
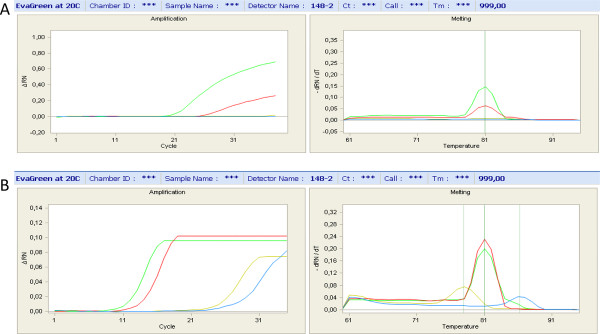
**Genotyping by Fluidigm BioMarkTM IFC Dynamic ArrayTM quantitative PCR.(A)** WGA-FLDMqPCR: WGA-amplified DNA and qPCR. Left: double-strand DNA (dsDNA) accumulation curve as a function of the number of cycles. Right: melting curve of the final product. Green: positive control (duck DNA). Red: a hybrid which was positive (containing duck DNA corresponding to the marker tested). Blue: a negative hybrid. Yellow: negative control (hamster DNA). **(B)** Pre-ampFLDMqPCR: non-amplified DNA, a pre-amplification step with a mix of the 96 primer pairs for the 96 markers tested in the Fluidigm BioMarkTM assay and qPCR. The same markers and controls are used as in **(A)**. The sensitivity is higher in **(B)**, with a lower number of cycles necessary for detection of duck DNA. The negative control and the hybrid not containing duck DNA amplify at a much higher number of cycles and the non-specific products amplified can easily be distinguished by their different melting temperature values (right). In both experiments, no amplification was obtained from water (data not shown).

However, genotyping several thousand markers by individual PCR and gel electrophoresis would require a lot of time and effort and a higher throughput method would be more appropriate, if feasible. In addition to scaling down PCR volumes and reducing required DNA amounts, the Fluidigm BioMark^TM^ has the added benefit of allowing rapid testing of 96 markers on 96 samples. To compare Fluidigm BioMark^TM^ genotyping by qRT-PCR, with (WGA-FLDMqPCR) or without (Pre-ampFLDMqPCR) WGA of the radiation hybrid DNA (see methods), with our more usual PCR and agarose (WGA-DNA) method, we used a set of 39 markers designed from scaffolds of the duck genome assembly. Results shown in Figure 1B suggest differences in retention frequencies between the three methods for the 39 markers tested, with lower values for the WGA-FLDMqPCR method. Differences in marker retention between the three methods were estimated by multiple t-tests (Table 1 and Additional file 1: Table S1), suggesting that there was no significant difference between the WGA-PCR and the Pre-ampFLDMqPCR methods, whereas the WGA-FLDMqPCR genotyping results were significantly different from the two others, with markedly lower retentions. These lower retentions values found with the WGA-FLDMqPCR condition are probably due to a lower sensitivity of the method, when compared to the Pre-ampFLDMqPCR condition (Figure 2). Taken together, our results suggest that our genotyping method by qPCR using the Fluidigm BioMark^TM^ IFC Dynamic Array^TM^ should not be performed using WGA DNA and also that if WGA DNA can be used for genotyping macrochromosome markers by the conventional agarose technique, it may cause problems for the smallest microchromosomes, as suggested by the results from the two markers on APL17.

**Table 1:** Comparison of marker retention with the three genotyping techniques

	**WGA- PCR**	**WGA- FLDMqPCR**	**Pre- ampFLDMqPCR**
**WGA-PCR**	**26.1**	15.9	24.8
**WGA-FLDMqPCR**	7.4e-08	**16.2**	15.8
**Pre-ampFLDMqPCR**	0.7	2.1e-10	**28.1**

Diagonal (in bold): mean number of positive hybrids in the panel (90 hybrids; 39 markers tested). Above the diagonal: mean number of positive hybrids in common between two conditions. Below the diagonal: P-values adjusted by Bonferroni correction for the differences in marker retention between two techniques.

To investigate further the possibility of using the Pre-ampFLDMqPCR method for genotyping, we constructed a map for a medium-size microchromosome (APL12), in addition to a first map of APL22 constructed by conventional PCR and agarose genotyping.

### RH mapping of APL22 by WGA-PCR

**Figure 3:**
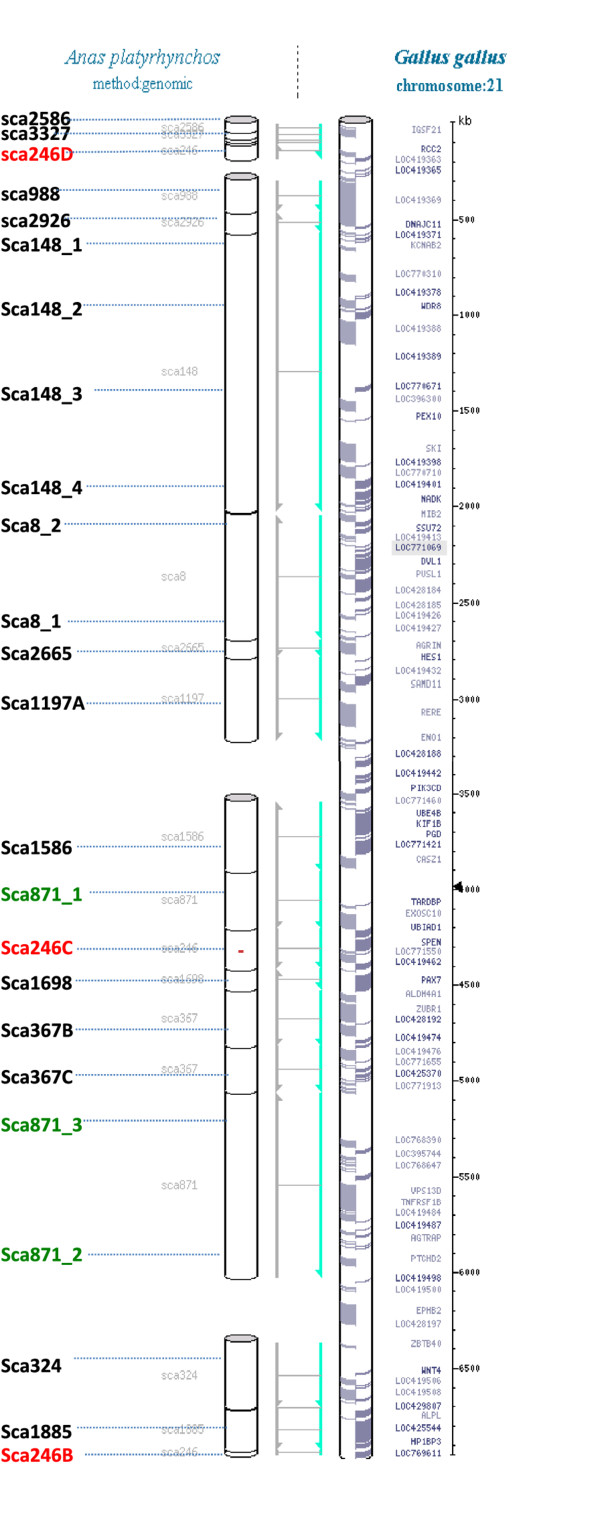
**Developing markers using comparative mapping data.** Screenshot of GGA21 from the Narcisse database (http://narcisse.toulouse.inra.fr/pre-narcisse/duck/cgi-bin/narcisse.cgi). Right: GGA21, with gene names. Left: white cylinders represent duck scaffolds or portions of duck scaffolds aligned to the chicken genome. Grey and green arrows represent portions of conserved synteny between the chicken chromosome and the duck scaffolds and their orientation. Left: names of the markers developed for RH mapping. For large scaffolds, such as sca148, one marker every 500 kb was developed to ensure RH linkage by optimizing inter-marker distances. Red: scaffold246 and green: scaffold871. These two scaffolds each seem to be split in chicken into three and two different regions respectively. At least one marker per region was developed, so as to check duck scaffold integrity.

**Figure 4:**
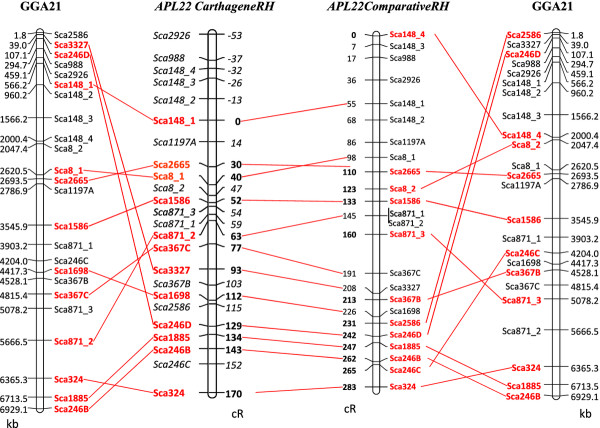
**Comparative mapping between chicken chromosome 21 (GGA21) sequence map and duck chromosome 22 (APL22) radiation hybrid maps.** Left and right: position of duck scaffold markers on the chicken genome. Middle left: RH map built with the Carthagene software. Middle right: RH map built with the comparative approach, followed by statistical confidence measures for genome maps. Framework markers for the CarthageneRH map and robust markers for the ComparativeRH map are in red.

Twenty-four scaffold markers derived from 15 duck scaffolds aligned to GGA21 in the Narcisse database (Figure 3) and designed as described in the Material and Methods section, were genotyped on WGA DNA by conventional PCR and gel electrophoresis. To build a RH map of microchromosome APL22, two methods were used: one using the Carthagene software with the usual method [41] and a second using a comparative approach based on the chicken genome, and the construction of a robust map (see Methods). By the classical Carthagene approach, 24 markers were included in a single linkage group with a LOD score threshold of 11, and a framework map containing 12 markers and spanning 170 cR was obtained. However, five of the comprehensive map markers might extend the current map length by 53 cR and the most likely position for all framework and non-framework markers given by the Carthagene software [41] are indicated in italics on the APL22 RH map (Figure 4). The comparative mapping approach and the associated robust map construction produced a map 283 cR long, containing 12 robust markers (Figure 4). The average retention frequency for the markers is 30.4%, in accordance with microchromosome retention frequency of the panel. A maximum marker retention around marker sca246B, suggests the centromere could be in that region (data not shown), which would be compatible with an acrocentric microchromosome. Comparative mapping with chicken chromosome GGA21 suggests several intrachromosomal rearrangements within this microchromosome.

### RH mapping of APL12 by Pre-ampFLDMqPCR

**Figure 5:**
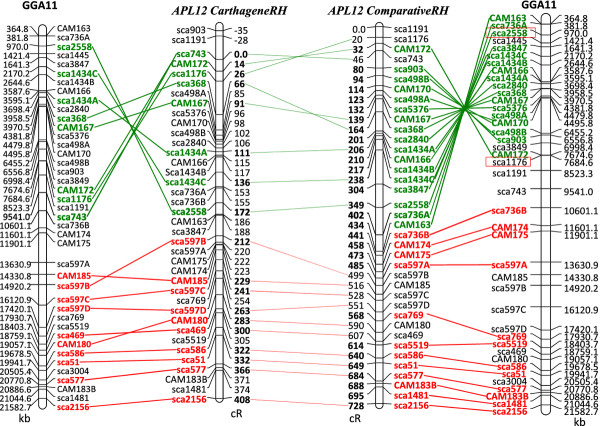
**Comparative mapping between chicken chromosome 11 (GGA11) sequence map and duck chromosome 12 (APL12) radiation hybrid maps.** Left and right: position of duck scaffold markers on the chicken genome. Middle left: RH map built with the Carthagene software. Middle right: RH map built with the comparative approach, followed by statistical confidence measures for genome maps. Framework markers for the CarthageneRH map and robust markers for the ComparativeRH map are in red or in green (inversion). Two markers boxed in red correspond to the chicken BAC clones used for FISH mapping.

Genotyping data for ten APL12 microsatellites and thirty-one markers designed from 18 scaffolds aligned to GGA11 were successfully obtained using the Pre-ampFLDMqPCR method and used to generate a RH map by the classical approach with the Carthagene software [41] and by the comparative mapping approach. After two-point analysis at a LOD threshold of 6, three linkage groups were defined among which the largest one contained 38 markers. The order of the 38 markers from the largest linkage group was determined by multipoint analysis with Carthagene and a framework map of APL12 bearing eighteen markers was obtained. The framework map is composed of 18 markers, covers 408 cR_6000_ and twenty additional markers on the comprehensive map extend the current map length by 34 cR (Figure 5). The map obtained by the comparative mapping approach is 728 cR long. The average retention for the markers on APL12 is 46%, significantly higher than the average microchromosome retention. As a result, the whole chromosome has a relatively high retention rate and no position for the centromeric region can be suggested from the RH map.

**Figure 6:**
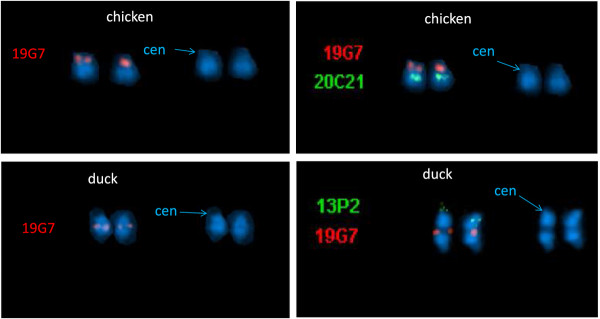
**Confirmation of the inversion on APL12 by FISH.** Chromosomes are stained by DAPI**.** Centromere positions (cen) are indicated by arrows. Left: BAC clone WAG19G7, corresponding to duck scaffold sca2558 is located in the centromeric region of GGA11 (top), whereas it is clearly located in the middle of the q arm of APL12 (bottom), suggesting the occurrence of an intrachromosomal rearrangement. Right: BAC clone WAG19G7 (red) corresponds to scaffold2558, whereas WAG20C21 and WAG13P2 (green) to scaffold1176. In chicken WAG19G7 (scaffold2558) is located in the centromeric region of GGA11 and WAG20C21 (scaffold1176) is in the middle of the q arm (top), whereas in duck, WAG19G7 (scaffold2558) is located in the middle of the q arm and WAG13P2 (scaffold1176) is at the end (bottom). This suggests the occurrence of an inversion between the two species. The black bands in the middle of APL12 near BAC clone 19 G7, might be an artifact resulting from over-denaturation or to the DAPI staining.

Only one major intrachromosomal rearrangement is observed when comparing APL12 to chicken chromosome GGA11. One additional minor inversion is observed only when comparing GGA11 with the map of APL12 built with the classical Carthagene approach. The major inversion was tested and confirmed by FISH mapping using BAC clones located at both ends of the inverted fragment and corresponding to the regions of scaffold2558 and scaffold1176 (Figure 5). FISH results confirm the inversion (Figure 6).

### RH Mapping of *no hit* EST markers from the smallest microchromosomes

Next, we wanted to test the three genotyping methods for mapping the smallest microchromosomes, orthologous to those absent from the current chicken sequence assembly and maps. We previously reported a strategy for mapping genes on the smallest microchromosomes absent from the chicken genome assembly [42,43]. Chicken EST contigs with sequence similarity to the human genome and showing no BLAST hit in the chicken genome were selected to develop PCR markers. Most of these markers, which we named the *no hit* markers (see materials and methods), were found to cluster in specific regions of the human genome, likely corresponding to conserved syntenies missing in chicken and corresponding to the missing microchromosomes [42].

**Figure 7:**
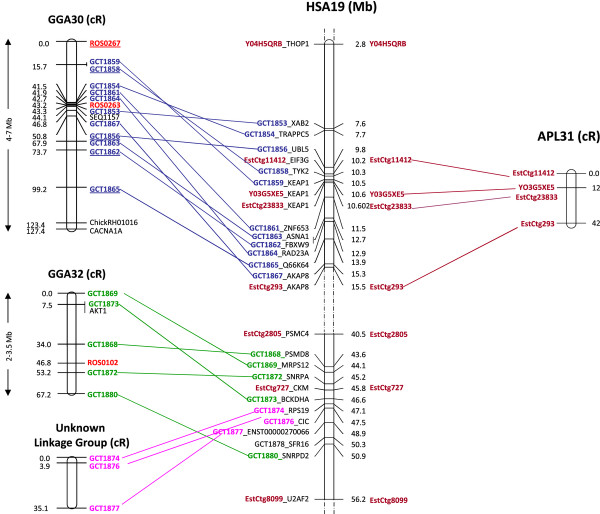
**Chicken and duck microchromosome linkage groups based on ‘no hit’ EST mapping. ***-Left* : the chicken linkage groups are from Morisson *et al.,* 2007 [43]. Markers were developed from chicken EST contigs absent from the chicken assembly (*no hit* markers), presenting sequence similarity to HSA19. Markers in blue, purple or green are ‘*no hit*’ EST; genetic markers are in red and framework markers are underlined. Markers in black got subsequently included in the linkage groups.*-Middle:* position on HSA19 of chicken EST markers (blue, purple or green) and duck EST markers (brown). For each marker, the name of the gene is added. The duck EST markers are shown on both sides of the map to allow visualization of all possible pair wise map comparisons.*-Right:* a duck RH linkage group corresponding to one part of chicken microchromosome GGA30. They both bear the genes AKAP8 (GCT1867 in chicken and EstCtg293 in duck) and KEAP1 (GCT1859 in chicken and Y03G5XE5, EstCtg23833 in duck). Markers were developed from duck EST contigs, presenting sequence similarity to HSA19 and for which no sequence similarity could be found on the chicken genome.

To increase chances of our markers showing linkage in duck, we focused marker development on duck EST contig sequence having sequence similarity to HSA19, in a region that was already shown to have synteny conservation with some of the smallest chicken chromosomes and being absent in the chicken genome assembly [43]. Due to the limited amount of duck EST data available, we were able to design only eight such markers derived from duck EST contig data showing no significant BLAST hits with the current chicken assembly (chicken *no hit* markers) but with sequence similarity to human chromosome HSA19 (Figure 7). These were genotyped by all three techniques. Genotyping results for these eight *no hit* to chicken markers are showed in Table 2, suggesting WGA has a much lower efficiency for the smallest microchromosomes, especially when used in combination with the FLDM method, leading to the underrepresentation (much lower retentions) or even to the total loss of the corresponding mapping data. For instance, the genotyping results of the 8 *no hit* markers showed that some regions like the fragment spanned by marker *EstCtg23833* are not amplified by WGA because no positive signal was observed both in amplified hybrid DNA or in amplified duck genome DNA, whereas the remaining seven markers have a very low average retention: 5% in WGA-FLDMqPCR and 12% in WGA-PCR, compared to 34% for Pre-ampFLDMqPCR. The latter method seems thus the only one suited for mapping the smallest microchromosomes.

**Table 2:** **Genotyping 8 *no hit *markers using three different genotyping strategies**

	**WGA-PCR**			**WGA-FLDMqPCR**			**Pre-ampFLDMqPCR**		**BLAST to duck assembly**	
	**WGA Duck**^**1**^	**Duck**^**2**^	**No. pos**^**3**^	**WGA Duck**^**1**^	**Duck**^**2**^	**No. pos**^**3**^	**Duck**^**2**^	**No. pos**^**3**^	**BLAST hit (scaffold name)**	**Scaffold length (bp)**
**EstCtg11412**	+	+	11	+	+	3	+	25	sca4924	26 914
**EstCtg23833**	-	+	0	-	+	0	+	25	C19155564	548
**EstCtg2805**	+	+	18	-	+	3	+	24	sca12946	245
**EstCtg293**	+	+	24	+	+	16	+	30	sca271	23 394
**EstCtg727**	+	+	14	-	+	1	+	44	nohit	NA
**EstCtg8099**	-	+	1	-	+	2	+	29	C18154597	159
**Y03G5XE5**	+	+	7	-	+	0	+	25	nohit	NA
**Y04H5QRB**	+	+	13	+	+	11	+	43	sca1017	95 902
**Nb. Controls**^**4**^**or Mean pos**^**5**^	6/8	8/8	11	3/8	8/8	4.5	16/16	30.6	NA	NA
**Mean retentions (%)**	NA	NA	12	NA	NA	5	NA	34	NA	NA

The 8 *no hit* markers were genotyped using either of three methods (see Material and Methods): (i) WGA-PCR: the WGA-amplified panel and conventional agarose genotyping; (ii) WGA-FLDMqPCR: the WGA-amplified panel and genotyping with the Fluidigm BioMark gene expression dynamic array, without the pre-amplification with a mix of all primer pairs or (iii) Pre-ampFLDMqPCR: the non-amplified panel and genotyping with the Fluidigm BioMark^TM^ IFC Dynamic Array^TM^ with a pre-amplification step using a mix of all primer pairs. ^1^WGA Duck: WGA-amplified duck genomic DNA as positive control; ^2^Duck: duck genomic DNA as positive control; ^3^No. Pos: number of hybrids positive for the assay (out of 90 hybrids tested); ^4^ Nb. Controls: total number of controls which are positive over the number of controls tested; ^5^Mean pos: mean number of positive hybrids observed over the whole panel; *NA*: not applicable.

Analysis of the results with Carthagene showed that 4 out of the 8 markers: *EstCtg11412*, *EstCtg23833, YO3G5XE5* and *EstCtg293* are linked together and define a region of conserved synteny with HSA19 and GGA30 (Figure 7) and corresponds thus to APL31. The duck marker *EstCtg727* labels the gene *CKM* which is located very close to human genes *BCKDHA, SNRPA, MRPS12* and *PSMD8* which were shown to be on GGA32. This suggests that *EstCtg727* could be located on duck chromosome APL33.

### Testing scaffold assembly

To test the quality of scaffold assembly, we selected the 13 largest duck scaffolds whose length ranged from 4.0 to 5.9 Mb and designed 70 markers with a density of one marker every megabase. These 70 markers were genotyped by Pre-ampFLDMqPCR and the results allowed the detection of one possible misassembly in scaffold504, for which a marker located at one end showed no linkage with the others. Results for all the scaffolds are showed in Additional File 2: Figure S1.

**Figure 8:**
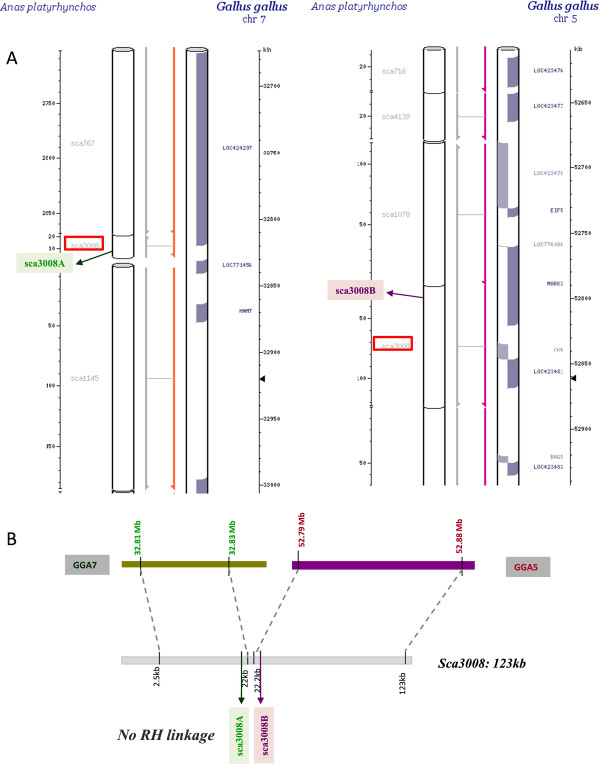
**Testing duck scaffolds aligning to two chicken chromosomes.** Based on previous observations, duck scaffolds aligning to two chicken chromosomes were suspected to be misassembled and one example is shown here. A: sca3008, boxed in red, aligns to GGA5 and GGA7, according to the Narcisse database. B: Markers sca3008A (green) and sca3008B (purple), very close to one another on sca3008, but spanning the putative breakpoint, were genotyped on the RH panel, but failed to show linkage, indicating that the scaffold is indeed misassembled. Results for other scaffolds are shown in Additional File 3: Figure S2.

To further test scaffolds from the duck genome assembly, we screened for potential chimeras by comparative mapping and detected using Narcisse [32], 41 duck scaffolds each of which mapping to two chicken chromosomes (Figure 8). As no inter-chromosomal rearrangements have been described to date between duck and chicken, we suspected assembly errors could have occurred and therefore tested 19 of the breakpoints by RH mapping with 45 markers. Results showed that all scaffolds, with the notable exception of sca649 could be misassembled (Figure 8 and Additional File 3: Figure S2).

## Discussion

Overall, the pattern of retention for the broken duck chromosome fragments in the hamster cells obtained here is very similar to that observed for the chicken radiation hybrid panel, with higher retentions for microchromosomes than for macrochromosomes. However, whereas only 23% of the chicken-hamster hybrids produced had sufficient retention frequency values to be retained in the final panel, 50% of the duck-hamster clones did. Indeed, although the fusion efficiency for chicken-hamster hybrids was reported to be as high as 2–9 x 10^-6^ by Kwok *et al.*[44], it was only approximately 1.4 x 10^-6^ in our hands when we produced the 452 clones for the chicken whole genome RH panel. Here, the fusion efficiency is close to 3.5 x 10^-6^ which is three times higher. Such differences could be due to variations in chromosome structure and/or content between the two bird species or to differences in culture conditions. For instance, the *HPRT* gene used as a selection marker for the clones is on the short arm of macrochromosome GGA4 in chicken [45] and thus very likely to be on microchromosome APL10 in duck. Microchromosomes being better retained than the macrochromosomes, having the selection gene on one of them could help recovering a higher number of clones in each fusion experiment. It is also very likely that these results are due to our change in culture conditions after the cell fusions: the chicken-hamster hybrids were cultivated at 40°C, the usual temperature for avian cells, whereas the duck-hamster ones at 37°C. Similarly, Ekker *et al.*[46] succeeded in producing zebrafish somatic hybrids at 37°C but not at 28°C, which is the normal temperature for the culture of zebrafish cells. More generally, the difference in optimal temperatures for the growth of donor and recipient cells may be one of the possible causes for the lower retention frequencies usually observed for somatic and radiation hybrids in non mammalian species.

To obtain the DNA quantity required for building genome-wide maps, large-scale culture of the hybrid clones is necessary. However, in this process, donor DNA is lost. For instance, Karere *et al.*[47] reported a genome wide half-life of the donor DNA of 8.7 passages and when preparing the whole genome RH (WGRH) panel in chicken, we observed the loss of 10% of the chicken genome after large cell culture of the hybrids [38]. This problem, in addition to the fact that large-scale culture of a RH panel requires lots of labor, encouraged us to find an alternative, such as WGA or scaling down the reaction volumes. Since the 1990s three major whole genome amplification techniques including primer extension pre-amplification (PEP) [48], degenerate oligonucleotide primed (DOP) PCR [49] and multiple displacement amplification (MDA) have been developed to address the problem of limiting amounts of DNA samples. PEP and DOP are both PCR-based methods and are limited by features of the Taq polymerase: typical amplification fragment length of < 3 kb and an error rate of 3 × 10^-5^. These methods also suffer from incomplete coverage and uneven amplification of the genomic loci of several orders of magnitude, with 10^-2^ ~ 10^-4^ and 10^-3^ ~ 10^-6^ fold amplification biases for PEP and DOP-PCR methods, respectively [50]. MDA is an isothermal amplification employing the high fidelity Phi29 phage DNA polymerase for DNA synthesis and strand displacement [51]. The genome coverage is much improved, with an estimation of only 2.2% missing after WGA by the MDA method in mammals [52]. Karere *et al.*[47] confirmed that MDA was suitable for RH mapping and reported a high concordance rate of 97.6% with data from genomic DNA. However, even if this is true for mammals, it might not be the case of microchromosomes in an avian genome.

When comparing retention frequencies before and after WGA in the 90 hybrids, with the 35 markers used for clone selection, only slight variations of retention, either gains or losses, were usually observed. However three markers, *CAUD064, S618* and *CAUD022,* show an important loss of retention frequency after WGA while two others, *CAUD013* and *S2870*, show a high increase, suggesting potential coverage problems by the WGA, either by lack of coverage (losses) or by the over-representation of a region (gains). Moreover, genotyping of eight *no hit* EST markers on WGA DNA, either using conventional PCR and Agarose or FLDMqPCR*,* demonstrated a very low retention which is not in accordance with the retention levels usually observed for microchromosomes. Therefore, we suggest that the genomic features in the smallest microchromosomes causing coverage problems in whole genome sequencing projects may also interfere with the efficiency of WGA. As we have already shown, RH mapping can allow building maps for non-sequenced chromosomes [42,43], it is important that we produce genotyping results for them.

In this context, the Fluidigm BioMark^TM^ IFC Dynamic Array^TM^ genotyping method can be an alternative to WGA, as only minute amounts of DNA (as little as 70 pg) are required. High throughput gene expression analysis by real time PCR in a microfluidic dynamic array was first introduced by Spurgeon *et al.*[40], and has since been successfully applied to copy number variation studies [53] and quantitative miRNA expression analysis [54]. In our case, by performing qPCR with the Fluidigm BioMark^TM^ IFC Dynamic Array^TM^ genotyping, the additional benefit is high throughput, as the identification of bands on gel electrophoresis is replaced by monitoring the PCR with Ct (Cycle threshold) and end point Tm (melting temperature) values, allowing the distinction between specific and non-specific amplification profiles. The Tm value is mainly influenced by base composition of amplicons, making it a specifically interesting parameter to follow when using markers defined from coding regions, which are more prone to cross-amplifying the hamster DNA.

We tested the Fluidigm genotyping method on WGA DNA and on standard DNA, with a pre-amplification step using a mix of all primers of the 96 markers analyzed together in a run [55]. In the WGA-FLDMqPCR runs, Ct values for the duck positive control was high with an average of 22 cycles (data not shown), as opposed to an average of 12 cycles, which is in the recommended scale, for the Pre-ampFLDMqPCR runs (Figure 3). These high Ct values suggested the quantity of DNA template was too low [55]. For a variety of reasons, WGA coupled with either FLDMqPCR or conventional PCR and agarose electrophoresis was unsuitable for genotyping on the smallest microchromosomes. Therefore, although the combination of WGA and FLDMqPCR would have allowed us to use less RH DNA, we decided the best genotyping method was to use standard DNA by FLDMqPCR genotyping, with a pre-amplification step performed using a mix of all primers for a set of 96 markers.

The drawbacks of genotyping by Fluidigm BioMark^TM^ IFC Dynamic Array^TM^ come from the fact that all 96 markers are genotyped with the same condition and therefore special care must be taken in the marker design. As a consequence, approximately 10% of the markers were discarded during the final analysis due to poor quality data.

Apart from improving the genome assembly by assigning and ordering scaffolds to chromosomes, the duck RH panel can be used to test the scaffold assemblies. We tested this by genotyping markers at Mb density on the 13 scaffolds larger than 4 Mb, spanning altogether 60 Mb and thus accounting for 5.5% of the current duck genome assembly. A total of 70 markers were genotyped and only one marker (sca504F) on the end of *sca504* was not linked with other markers derived from the same scaffold (Additional File 2: Figure S1), suggesting an overall good quality of the final genome. To test further our capacity for detecting potentially misassembled scaffolds, we took advantage of previously published data indicating that on the whole, avian chromosomes are known to be well conserved throughout evolution and more specifically, that no inter-chromosomal rearrangements, apart from the well documented case of GGA4 = APL4 + APL10, have been discovered between chicken and duck by current comparative cytogenetic approaches [17,23,28]. The 41 scaffolds (including sca504) we detected as potential chimeras by comparative mapping had poor pair-end sequence support (BGI, personal communication), suggesting most of them could indeed be misassembled (Additional file 4: Table S2). We tested nineteen of them by genotyping markers flanking the potential breakpoints (Additional File 3: Figure S2). As a result, all but one scaffold (*sca649*) could be misassembled, and *sca649* possibly suggesting the first detection of a small inter-chromosomal rearrangement between the duck and chicken genomes, or perhaps more likely a segmental duplication in duck or in the last common ancestor of the two species. This would need further confirmation by FISH mapping with chicken BAC clones. It can be noted that the pair-end sequence support for this scaffold was high, showing an agreement between sequencing and RH mapping data. When disagreements between assembly and our RH data are detected in large scaffolds, they tend to happen towards the end. To achieve better assembly accuracy, higher sequencing depth or more efforts on developing sequencing libraries with longer inserts are needed.

Concerning the smallest duck microchromosomes, paralogous to those absent from the chicken assembly, we suspect similar problems will arise: lack of sequence information, difficulties in cloning, in genetic mapping, etc. RH mapping has proved useful for getting a grip on these regions and one striking example is the case of some regions of HSA19, to which no corresponding chicken genome data could be assigned by sequence similarity and to which many chicken *no hit* EST showed significant sequence similarity. RH mapping with these markers allowed building maps for GGA30 and GGA32 [43]. By developing markers targeted to this region, a small linkage group composed of 4 *no hit* markers (absent in the chicken genome assembly) orthologous to HSA19 was obtained. When aligned to HSA19, we found they spanned a 5 Mb region on HSA19p. Due to the lack of BAC clones for FISH or other supplementary information, we cannot identify the duck chromosome, but according to known data on synteny conservation between chicken and duck, we suggest that this small linkage group should be assigned to APL31. Of the 8 *no hit* to chicken markers we studied three have hits with small to medium-size scaffolds (between 23 and 96 kb) of the duck assembly, suggesting that more sequence from the smallest microchromosomes could be obtained in NGS (Table 2). Chicken/duck comparative mapping of GGA21/APL22 and GGA11/APL12 microchromosomes demonstrate several intrachromosomal rearrangements, the first described for microchromosomes in this pair of species. The maps obtained using the usual Carthagene mapping approach or the comparative approach are very similar, apart for a few markers, especially non-framework / non-robust ones, for which lower reliability in map position can be due to the limits of the possible resolution of the mapping or to genotyping errors. As the comparative approach starts with an ordering of markers corresponding to chicken, it is interesting to note that the major duck-chicken rearrangements found with the Carthagene approach are confirmed. A second advantage of the comparative mapping approach and the associated construction of robust maps is that the number of robust markers obtained is usually higher than the number of framework markers in the classical approach. The major inversion found between GGA11 and APL12 is confirmed by FISH mapping, but also by sequence alignment of duck scaffolds on the chicken assembly. Indeed, scaffold736, whose integrity is demonstrated by RH mapping, with markers *sca736A* and *sca736B* positioned close to one another at 153 and 154 cR on the CarthageneRH map and 402 and 441 cR on the ComparativeRH-Robust map, is separated in two locations when aligned to the chicken sequence (Figure 5). Likewise, although a more complex pattern of events accounts for the differences between GGA21 and APL22, one of them is supported by scaffold246, whose integrity is demonstrated by RH mapping with three markers on the robust map, each of which are positioned in different regions when aligned to the chicken sequence. Another is supported by markers sca871_1 and sca871_2, which are co-localized on the RH map and are 1.4 Mb apart in chicken (Figures 3 and 4). When comparing the turkey and chicken genomes, Zhang *et al.* also confirmed evidence for 20–27 major rearrangements between the two species and found one inversion between GGA11 and MGA13. However, they did not observe any rearrangement between GGA21 and MGA23. The mean estimated divergence time between chicken and turkey is 47 million years and 81 between chicken and duck [56]. A higher number of rearrangements are thus expected between the two latter pair of species. Only one major interchromosomal difference -with APL4 and APL10 corresponding to GGA4q and GGA4p respectively [21]- and very few intrachromosomal rearrangements have been reported between the chicken and the duck karyotypes [18-23]. The rearrangements observed with our data between GGA21 and APL22 seem more complex for example, Sca246B, Sca246C and Sca246D are split by Sca1885 in both RH maps. Likewise, Sca367B and Sca367C are split by Sca3327 in both RH maps, whereas they are adjacent in the chicken sequence, and Sca148 markers are widely split in the ComparativeRH map, while adjacent in the chicken. Further investigations and more precise maps using different techniques such as FISH or BAC contig maps will be needed to confirm these rearrangements. The increased resolution obtained by RH mapping as compared to the FISH mapping performed to date show that intrachromosomal rearrangements might happen on a finer scale than shown until now. This means that although the simple ordering of the duck scaffolds along the chicken genome by sequence similarity helps for chromosome assignment, the duck sequence thus obtained will be wrong whenever large or small-scale rearrangements will have happened between the two species. The whole duck assembled sequence will have to be ordered using the whole genome RH map which will be constructed in our laboratory, in conjunction with other mapping methods, such as genetic and/or BAC contig physical maps, the latter allowing finer mapping and orientation of small scaffolds.

## Conclusion

The chicken WGRH panel has been used to construct chromosome RH maps and helped in the genome assembly or the mapping of some of the smallest micro chromosomes [42,43,57]. Similarly, the duck WGRH panel presented here will also be a major contribution to duck genomics. RH mapping can be a complementary approach to NGS by allowing the assignment of scaffolds to duck chromosomes and furthermore, detailed RH maps will allow a precise estimation of the intrachromosomal rearrangements that have occurred between chicken and duck.

Using the chicken genome as model and in combination with survey sequencing, the construction of dense RH maps of a less studied bird such as duck can be made. By taking advantage of the conservation of syntenies, optimal orders can be proposed [58,59], thus maximizing the information obtained as first proposed by Hitte *et al.*[60,61]. Indeed in duck, a dense RH map combined with scaffold sequencing and comparison to the chicken sequence, should lead to an improved genome assembly.

## Material and methods

### Generation of radiation hybrids

The method was adapted from Morisson *et al.* 2002 [38]. Normal diploid fibroblasts were obtained from 12-day-old Peking duck embryos from a highly inbred duck line. For each embryo, primary cells were obtained after trypsinization of the embryo tissues and the rest of the tissues were stored for DNA extraction. Duplex PCR was performed to test the sex of embryos according to Batellier *et al.*[62]. Fibroblasts from only one female embryo were propagated in complete DMEM medium (DMEM Glutamax (Gibco Co.) supplemented with 10% foetal calf serum (Gibco Co.), 1% penicillin and streptomycin) at 40°C with 5% CO_2_ and used as donor cells. The *HPRT* (Hypoxanthine guanine phosphoribosyl transferase) -deficient hamster cell line Wg3hCl_2_[63] was used for recipient cells, which were cultured in complete RPMI medium (RPMI1640 (Sigma Chemical Co.) supplemented with 10% foetal calf serum (Gibco Co.), 1% penicillin and streptomycin) at 37°C with 5% CO_2_. For each fusion experiment, 1.5 × 10^7^ duck female fibroblasts were irradiated at 6000 rads by gamma rays from a Cesium-137 source and mixed to an equal number of Wg3hCl_2_ hamster cells. The fusion partners were then pelleted and suspended in 1 mL polyethyleneglycol (Roche Diagostics GmbH) and after 1 min, 15 mL DMEM medium without serum and antibiotics were gradually added from which 1 mL was taken to suspend in 10 mL complete RPMI medium and cultured at 37°C with 5% CO_2_. Twenty-four hours after the fusion, HAT (hypoxanthine-aminopterin-thymidine) was added to the medium and four days later, the whole medium was changed to discard the non-fused cells. Eight to twelve days after the fusion, the first hybrid clones were observed. When fully grown after 7 to 20 days of culture, hybrids were picked and transferred to 25-cm^2^ flasks. After confluence, the hybrid cells were subsequently transferred to two 75-cm^2^ flasks. In order to limit the loss of duck fragment during the cell passages, hybrids were cultured for only one generation and harvested when fully grown. Ten million cells were kept for DNA extraction and the rest were cryoconserved.

### Whole genome amplification

For each sample, 50 ng starting RH DNA was amplified according to the GE Healthcare Illustra Genomiphi HY DNA amplification Kit protocol. To avoid representation bias, each hybrid was amplified in three replicates which were pooled to obtain the final working panel DNA (WGA DNA). Duck genomic DNA, Wg3hCl_2_ hamster DNA and H_2_O were amplified in the same condition, as positive and negative controls. For genotyping experiments with the WGA DNA, the positive controls were duck genomic DNA and WGA duck genomic DNA, whereas when using standard DNA, both positive controls were duck genomic DNA.

### *In silico* mapping of scaffolds to the chicken assembly, as a guide for choosing markers

Seven thousand two hundred and five duck scaffolds larger than 1 kb were aligned to the current chicken assembly using Narcisse [31] and 1,787 were successfully positioned. All the data can be traced back at http://narcisse.toulouse.inra.fr/pre-narcisse/duck/cgi-bin/narcisse.cgi. According to Narcisse and existing comparative genomics data obtained by FISH [23,28], approximate location of all the scaffold markers, especially chromosomal assignment could be inferred, but the real location still needed to be tested due to the possibilities of intrachromosomal rearrangement having occurred since chicken and duck divergence.

Thirty scaffolds were positioned on GGA11 and used for designing 31 potential APL12 markers, whereas 15 scaffolds were positioned on GGA21 from which 24 potential APL22 marker were derived.

### Markers design

Altogether, 234 markers were used in our study and detailed information is given in Additional file 5: and Table S3. Twenty one microsatellite markers are from public databases (markers *APHXXX, CAUDXXX, AMUXXX and APTXXX*) and 10 *CAMXXX* markers were produced by our laboratory (Marie-Etancelin *et al.*, in preparation); 8 EST markers (*EstCtg11412*, *EstCtg23833, YO3G5XE5, EstCtg8099*, *Y04H5QR8, EstCtg2805*, *EstCtg727*and *EstCtg293*) are from EST contig data (Pitel *et al.*, Huang *et al.*, in preparation); the rest of the markers (ScaXXX or SXXX) were designed from the sequence of duck scaffolds from the genome assembly (Huang *et al.*, in preparation) with the Primer3 software [64]. To avoid repetitive elements in the genome, the primers were checked by in-silico PCR [65] and the amplicon sequences aligned to the whole genome assembly by BLASTn.

### Marker genotyping by conventional PCR and agarose gel electrophoresis (WGA-PCR.)

PCR reactions contained 25 ng WGA DNA, 2 mM MgCl_2_, 0.5U Taq DNA polymerase (Promega Co.), 1X buffer (Promega), 200 μM of each dNTP, 0.15 μM of each forward primers, 0.2 μm of each reverse primers in a total volume of 15μL. PCR was performed on a GeneAmp PCR system 9700 thermocycler (Applied Biosystems): the first 5-min denaturation was followed by 48 cycles for microsatellite markers and 36 cycles for scaffold markers, each consisting of denaturation at 94°C for 30s, annealing at specific temperature for 30s and elongation at 72°C for 30s. PCR products were analyzed using a 2% agarose gel and were visualized by ethidium bromide staining. All the markers were genotyped in duplicate.

### Marker genotyping by Fluidigm BioMark^TM^ IFC Dynamic Array^TM^ quantitative PCR on WGA DNA (WGA-FLDMqPCR)

WGA DNA (90 panel samples, positive control: standard Duck DNA and WGA Duck DNA, negative control: standard Hamster DNA and WGA Hamster DNA, blank control: WGA H_2_O and H_2_O) and an assay set containing 96 primer pairs in which concentration of each primer pair is 20 μM were loaded on a Fluidigm BioMark^TM^ 96.96 Dynamic Array^TM^ IFC. WGA DNA was quantified by Picogreen, the ideal concentration of the DNA was of 50 ng/μL. In fact, WGA DNA proved difficult to quantify by Picogreen, likely due to the complex branched structure of the amplification product obtained. Real time PCR was performed in the presence of EvaGreen^TM^ DNA-binding dye, according to the manufacturer’s protocol [40]. All the markers were genotyped in duplicate.

### Marker genotyping by Fluidigm BioMark^TM^ IFC Dynamic Array^TM^ quantitative PCR on pre-amplified standard DNA (Pre-ampFLDMqPCR)

Standard DNA was quantified by Picogreen and diluted at a final concentration of 5 ng/μL. Primer pairs for the 96 markers included in one Fluidigm BioMark^TM^ run were diluted at a final concentration of 20 μM and distributed in a 96-well microplate called a 20 μM assay set. Then 8 μl of 0.1 M TE and 2 μl of each primer mix from the 20 μM set were pooled in a 1 mL Eppendorf tube and vortexed thoroughly (96 Markers Primer mix). Pre-amplification was performed in 5 μL, containing 2.5 μl Pre-amplification Master mix (Applied Biosystems), 1.25 μl of 96 Markers Primer mix and 1.25 μl DNA at 5 ng/μL (90 panel samples, positive control: genomic Duck DNA, negative control: genomic Hamster DNA, blank control: H_2_O ). After denaturation for 10 min at 95°C, a PCR was performed by 14 cycles of 15 s at 95°C and 4 min at 60°C, and a final elongation step at 20°C for 10 min. The pre-amplification products thus obtained were diluted 7 times before the Fluidigm BioMark^TM^ run. The 96 diluted pre-amplified samples and 20 μM 96 primer pairs assay set was loaded on a Fluidigm BioMark^TM^ 96.96 Dynamic Array^TM^ IFC, using the same procedure as for the WGA-FLDMqPCR marker genotyping method. All the markers were genotyped in duplicate.

### Interpretation of FLDM data

Data was analyzed using the Fluidigm Real-Time PCR Analysis software to obtain the Ct values (Cycle Threshold: number of cycles required for the fluorescent signal to cross a given threshold) and Tm values (DNA melting temperature which is influenced by the length and base composition of the DNA molecules amplified) (Figure 3). For the genotyping calling, the positive control (duck DNA) should not be too low or too high (Ct values between 10 and 16). A hybrid was called positive when the hybrid had a Ct value lower or equal to that of the negative control and a Tm value close to the positive control. A genotype was called “Unknown” when a hybrid had a high Ct value but the same Tm as the positive control or a low Ct value but a Tm value was slightly different (± 1.5°C) than the positive control. If no amplification of the positive control could be seen, the marker was discarded altogether.

### Map construction

Two methods for map construction were used: (i) a classical approach by two point and multipoint mapping, followed by the determination of the minimal set of markers for a framework map and (ii) a comparative map approach with statistical measure of a set of maps. The classical RH maps were constructed using the Carthagene software [41] in three steps: (1) linkage groups were defined by two point analysis using a LOD score threshold of 11 (for the RH map of APL22) or 6 (for the RH map of ALP12) (2) multipoint analyses were done to define a framework map for the larger linkage groups, using a LOD threshold of 3 for the framework maps (3) a comprehensive map was built by calculating the location of additional markers relative to the framework markers. The comparative map approach is described by Faraut *et al.*, (2007) [58]. It uses the information of marker adjacencies in a related genome, to assist the mapping process when the experimental data is not conclusive, thus directly producing comparative maps minimizing the number of breakpoints. The comparative mapping is followed by a statistical confidence measure of a distribution of maps to evaluate map uncertainties and produce a robust map, such as described in Servin *et al.* (2010) [66]. Finally the map figures were created using MapChart [67].

### Fluorescent *in situ* hybridisation (FISH)

Chicken BAC clones were chosen in the Wageningen BAC library [68] according to their known position, as estimated by BAC end sequence information, in regions paralogous to the breakpoint under study. WAG19G7 (accession number CZ566048) corresponds to the duck scaffold sca2558, while WAG13P2 (CZ561694) and WAG20C21 (CZ565661) correspond to sca1176. BAC clones were grown in LB medium with 12,5 μg/ml chloramphenicol. The DNA was extracted using the Qiagen plasmid midi kit.

FISH was carried out on metaphase spreads obtained from fibroblast cultures of 7-days old chicken and duck embryos, arrested with 0.05 μg/ml colcemid (Sigma) and fixed by standard procedures. The FISH protocol is derived from Yerle *et al.*, 1992 [69]. Two-colour FISH was performed by labelling 100 ng for each BAC clones with alexa fluorochromes (ChromaTide® Alexa Fluor® 488-5-dUTP, Molecular probes; ChromaTide® Alexa Fluor® 568-5-dUTP, Molecular Probes) by random priming using the Bioprim Kit (Invitrogen). The probes were purified using spin column G50 Illustra (Amersham Biosciences). Probes were ethanol precipitated, resuspended in 50% formamide hybridization buffer (for FISH on chicken metaphases) or in 40% formamide hybridization buffer for heterologous FISH. Probes were hybridised to chicken metaphase slides for 17 hours at 37°C and to duck metaphases for 48 H in the Hybridizer (Dako). Chromosomes were counterstained with DAPI in antifade solution (Vectashield with DAP, Vector). The hybridised metaphases were screened with a Zeiss fluorescence microscope and a minimum of twenty spreads was analysed for each experiment. Spot-bearing metaphases were captured and analysed with a cooled CCD camera using Cytovision software (Applied Imaging).

## Competing interests

The authors declare that they have no competing interests.

## Authors’ contributions

MR, SB and MM produced the RH panel. MR made the whole genome amplification, designed some markers, made and analyzed the genotyping. KF and EL assisted with marker genotyping and genotyping analysis. YH and NL provided the duck sequencing and assembly data. AV and MR built the RH map. VF performed the FISH mapping. TF made the alignment of the duck scaffolds to the current chicken assembly. MR, AV and MM drafted the manuscript. TF, AV and MM conceived the study, participated in its coordination and finalized the manuscript. All authors read and approved the final manuscript.

## Supplementary Material

Additional File 1 **Table S1.** Genotyping results of 39 scaffold markers and 8 no hit ESTs for the three different methods. The panel contained 90 hybrids. Click here for file

Additional File 2 **Figure S1.** Checking the 13 largest scaffolds by RH mapping. Each thick horizontal line represents a scaffold; arrows point to the names of the markers which were genotyped on the duck RH panel. The approximate position of the markers is shown as well as the scaffold lengths. Markers in the same color and contained within the same box are linked by RH mapping. For the 12 first scaffolds shown, the RH mapping data confirm the scaffold assembly. The last one, scaffold504, was the only one which was detected to be discontinuous, as marker sca504F is not linked by RH mapping to the five other markers sca504A, sca504B, sca504C, sca504D and sca504E. Comparative analysis with chicken shows that the portion of the scaffold containing sca504F aligns to GGA2, whereas the rest aligns to GGA1. Click here for file

Additional File 3 **Figure S2.** Testing duck scaffolds aligning to two chicken chromosomes. Duck scaffolds are represented together with the portions of chicken chromosomes to which they show high sequence similarity in the Narcisse database. The approximate position of the markers on the scaffolds and on the chicken genome is shown as well as the scaffold lengths To test if the synteny breakpoints are due to an evolutionary chromosomal rearrangement or a problem in the assembly of scaffolds, a pair of markers was chosen close together on the scaffolds, but spanning the break points. Whenever markers are linked together by RH mapping, they are contained in the same box and are represented in the same colour. Click here for file

Additional File 4 **Table S2.** The 41 disrupted scaffolds which could be aligned on two different chicken chromosomes. Break1: the right-most coordinate of the alignment of the left part of the scaffold to one chicken chromosome. Break 2: the left-most coordinate of the alignment of the left right part of the scaffold to another chicken chromosome. Chicken 1 and Chicken 2: chicken chromosomes to which the left and right parts of the duck scaffold align to respectively. Pair-end support: refers to the reliability of paired-end sequence data. Click here for file

Additional File 5 **Table S3.** Data on all markers genotyped in the study. Primer pairs, PCR conditions, and accession numbers (where applicable) are given. Click here for file

## References

[B1] Li R Zhu H Ruan J Qian W Fang X Shi Z Li Y Li S Shan G Kristiansen K De novo assembly of human genomes with massively parallel short read sequencing Genome Res 2010 20 2 265 272 10.1101/gr.097261.109 20019144 PMC2813482

[B2] Li R Fan W Tian G Zhu H He L Cai J Huang Q Cai Q Li B Bai Y The sequence and de novo assembly of the giant panda genome Nature 2010 463 7279 311 317 10.1038/nature08696 20010809 PMC3951497

[B3] Xia Q Guo Y Zhang Z Li D Xuan Z Li Z Dai F Li Y Cheng D Li R Complete resequencing of 40 genomes reveals domestication events and genes in silkworm (Bombyx) Science 2009 326 5951 433 436 10.1126/science.1176620 19713493 PMC3951477

[B4] Huang S Li R Zhang Z Li L Gu X Fan W Lucas WJ Wang X Xie B Ni P The genome of the cucumber, Cucumis sativus L Nat Genet 2009 41 12 1275 1281 10.1038/ng.475 19881527

[B5] Rubin CJ Zody MC Eriksson J Meadows JR Sherwood E Webster MT Jiang L Ingman M Sharpe T Ka S Whole-genome resequencing reveals loci under selection during chicken domestication Nature 2010 464 7288 587 591 10.1038/nature08832 20220755

[B6] Ye L Hillier LW Minx P Thane N Locke DP Martin JC Chen L Mitreva M Miller JR Haub KV A vertebrate case study of the quality of assemblies derived from next-generation sequences Genome Biol 2011 12 3 R31 10.1186/gb-2011-12-3-r31 21453517 PMC3129681

[B7] Dalloul RA Long JA Zimin AV Aslam L Beal K Blomberg Le A Bouffard P Burt DW Crasta O Crooijmans RP Multi-platform next-generation sequencing of the domestic turkey (Meleagris gallopavo): genome assembly and analysis PLoS Biol 2010 8 9 1000475 10.1371/journal.pbio.1000475 PMC2935454 20838655

[B8] Tuyen D The situation of duck production in Vietnam Proceedings of the International Seminar on Improved Duck Production of Small-scale Farmers in the ASPAC region 2007 123 133

[B9] Cheng Y Breeding and genetics of waterfowl Worlds Poult Sci J 2003 59 509 519 10.1079/WPS20030032

[B10] Marie-Etancelin CCH Brun JM Marzul C Mialon-Richard MM Rouvier R Genetics and selection of mule ducks in France: a review Worlds Poult Sci J 2008 64 2 187 207

[B11] Sturm-Ramirez KM Hulse-Post DJ Govorkova EA Humberd J Seiler P Puthavathana P Buranathai C Nguyen TD Chaisingh A Long HT Are ducks contributing to the endemicity of highly pathogenic H5N1 influenza virus in Asia? J Virol 2005 79 17 11269 11279 10.1128/JVI.79.17.11269-11279.2005 16103179 PMC1193583

[B12] Olsen B Munster VJ Wallensten A Waldenstrom J Osterhaus AD Fouchier RA Global patterns of influenza a virus in wild birds Science 2006 312 5772 384 388 10.1126/science.1122438 16627734

[B13] Gaidet N Cattoli G Hammoumi S Newman SH Hagemeijer W Takekawa JY Cappelle J Dodman T Joannis T Gil P Evidence of infection by H5N2 highly pathogenic avian influenza viruses in healthy wild waterfowl PLoS Pathog 2008 4 8 e1000127 10.1371/journal.ppat.1000127 18704172 PMC2503949

[B14] van Asseldonk MAPM Meuwissen MPM Mourits MCM Huirne RBM Economics of controlling avian influenza epidemics In Avian Influenza Prevention and Control, Springer Ed Series: Wageningen UR Frontis Series 2008 8 139 148

[B15] Kim JK NN Forrest HL Webster RG Ducks: the "Trojan horses" of H5N1 influenza Influenza Other Respi Viruses 2009 3 4 121 128 10.1111/j.1750-2659.2009.00084.x 19627369 PMC2749972

[B16] Burt DW Origin and evolution of avian microchromosomes Cytogenet Genome Res 2002 96 1–4 97 112 12438785 10.1159/000063018

[B17] Huang Y Zhao Y Haley CS Hu S Hao J Wu C Li N A genetic and cytogenetic map for the duck (Anas platyrhynchos) Genetics 2006 173 1 287 296 10.1534/genetics.105.053256 16510785 PMC1461431

[B18] Guttenbach M Nanda I Feichtinger W Masabanda JS Griffin DK Schmid M Comparative chromosome painting of chicken autosomal paints 1–9 in nine different bird species Cytogenet Genome Res 2003 103 1–2 173 184 15004483 10.1159/000076309

[B19] Derjusheva S Kurganova A Habermann F Gaginskaya E High chromosome conservation detected by comparative chromosome painting in chicken, pigeon and passerine birds Chromosome Res 2004 12 7 715 723 15505406 10.1023/B:CHRO.0000045779.50641.00

[B20] Kayang BB Fillon V Inoue-Murayama M Miwa M Leroux S Feve K Monvoisin JL Pitel F Vignoles M Mouilhayrat C Integrated maps in quail (Coturnix japonica) confirm the high degree of synteny conservation with chicken (Gallus gallus) despite 35 million years of divergence BMC Genomics 2006 7 101 10.1186/1471-2164-7-101 16669996 PMC1534036

[B21] Griffin DK Robertson LB Tempest HG Skinner BM The evolution of the avian genome as revealed by comparative molecular cytogenetics Cytogenet Genome Res 2007 117 1–4 64 77 17675846 10.1159/000103166

[B22] Griffin DK Robertson LB Tempest HG Vignal A Fillon V Crooijmans RP Groenen MA Deryusheva S Gaginskaya E Carre W Whole genome comparative studies between chicken and turkey and their implications for avian genome evolution BMC Genomics 2008 9 168 10.1186/1471-2164-9-168 18410676 PMC2375447

[B23] Skinner BM Robertson LB Tempest HG Langley EJ Ioannou D Fowler KE Crooijmans RP Hall AD Griffin DK Volker M Comparative genomics in chicken and Pekin duck using FISH mapping and microarray analysis BMC Genomics 2009 10 357 10.1186/1471-2164-10-357 19656363 PMC2907691

[B24] Warren WC Clayton DF Ellegren H Arnold AP Hillier LW Kunstner A Searle S White S Vilella AJ Fairley S The genome of a songbird Nature 2010 464 7289 757 762 10.1038/nature08819 20360741 PMC3187626

[B25] Volker M Backstrom N Skinner BM Langley EJ Bunzey SK Ellegren H Griffin DK Copy number variation, chromosome rearrangement, and their association with recombination during avian evolution Genome Res 2010 20 4 503 511 10.1101/gr.103663.109 20357050 PMC2847753

[B26] Zhang Y Zhang X O'Hare TH Payne WS Dong JJ Scheuring CF Zhang M Huang JJ Lee MK Delany ME A comparative physical map reveals the pattern of chromosomal evolution between the turkey (Meleagris gallopavo) and chicken (Gallus gallus) genomes BMC Genomics 2011 12 447 10.1186/1471-2164-12-447 21906286 PMC3189400

[B27] Hedges SB Dudley J Kumar S TimeTree: a public knowledge-base of divergence times among organisms Bioinformatics 2006 22 23 2971 2972 10.1093/bioinformatics/btl505 17021158

[B28] Fillon V Vignoles M Crooijmans RP Groenen MA Zoorob R Vignal A FISH mapping of 57 BAC clones reveals strong conservation of synteny between Galliformes and Anseriformes Anim Genet 2007 38 3 303 307 10.1111/j.1365-2052.2007.01578.x 17539975

[B29] Keightley PD Eyre-Walker A Deleterious mutations and the evolution of sex Science 2000 290 5490 331 333 10.1126/science.290.5490.331 11030650

[B30] van Tuinen M Hedges SB Calibration of avian molecular clocks Mol Biol Evol 2001 18 2 206 213 10.1093/oxfordjournals.molbev.a003794 11158379

[B31] Courcelle E Beausse Y Letort S Stahl O Fremez R Ngom-Bru C Gouzy J Faraut T Narcisse: a mirror view of conserved syntenies Nucleic Acids Res 2008 36 Database issue D485 490 17981845 10.1093/nar/gkm805 PMC2238891

[B32] Narcisse Duckhttp://narcisse.toulouse.inra.fr/pre-narcisse/duck/cgi-bin/narcisse.cgi

[B33] Kraus RH Kerstens HH Van Hooft P Crooijmans RP Van Der Poel JJ Elmberg J Vignal A Huang Y Li N Prins HH Genome wide SNP discovery, analysis and evaluation in mallard (Anas platyrhynchos) BMC Genomics 2011 12 150 10.1186/1471-2164-12-150 21410945 PMC3065436

[B34] Kraus RH Kerstens HH van Hooft P Megens HJ Elmberg J Tsvey A Sartakov D Soloviev SA Crooijmans RP Groenen MA Widespread horizontal genomic exchange does not erode species barriers among sympatric ducks BMC Evol Biol 2012 12 45 10.1186/1471-2148-12-45 22462721 PMC3364866

[B35] Yuan X Zhang M Ruan W Song C Ren L Guo Y Hu X Li N Construction and characterization of a duck bacterial artificial chromosome library Anim Genet 2006 37 6 599 600 10.1111/j.1365-2052.2006.01526.x 17121612

[B36] Lawrence S Morton NE Cox DR Radiation hybrid mapping Proc Natl Acad Sci USA 1991 88 17 7477 7480 10.1073/pnas.88.17.7477 1881887 PMC52323

[B37] James MR Richard CW 3rd Schott JJ Yousry C Clark K Bell J Terwilliger JD Hazan J Dubay C Vignal A A radiation hybrid map of 506 STS markers spanning human chromosome 11 Nat Genet 1994 8 1 70 76 10.1038/ng0994-70 7987395

[B38] Morisson M Lemiere A Bosc S Galan M Plisson-Petit F Pinton P Delcros C Feve K Pitel F Fillon V ChickRH6: a chicken whole-genome radiation hybrid panel Genet Sel Evol 2002 34 4 521 533 10.1186/1297-9686-34-4-521 12270108 PMC2705459

[B39] Barrett JH Genetic mapping based on radiation hybrid data Genomics 1992 13 1 95 103 10.1016/0888-7543(92)90207-9 1577497

[B40] Spurgeon SL Jones RC Ramakrishnan R High throughput gene expression measurement with real time PCR in a microfluidic dynamic array PLoS One 2008 3 2 e1662 10.1371/journal.pone.0001662 18301740 PMC2244704

[B41] de Givry S Bouchez M Chabrier P Milan D Schiex T CARHTA GENE: multipopulation integrated genetic and radiation hybrid mapping Bioinformatics 2005 21 8 1703 1704 10.1093/bioinformatics/bti222 15598829

[B42] Douaud M Feve K Gerus M Fillon V Bardes S Gourichon D Dawson DA Hanotte O Burke T Vignoles F Addition of the microchromosome GGA25 to the chicken genome sequence assembly through radiation hybrid and genetic mapping BMC Genomics 2008 9 129 10.1186/1471-2164-9-129 18366813 PMC2275740

[B43] Morisson M Denis M Milan D Klopp C Leroux S Bardes S Pitel F Vignoles F Gerus M Fillon V The chicken RH map: current state of progress and microchromosome mapping Cytogenet Genome Res 2007 117 1–4 14 21 17675840 10.1159/000103160

[B44] Kwok C Korn RM Davis ME Burt DW Critcher R McCarthy L Paw BH Zon LI Goodfellow PN Schmitt K Characterization of whole genome radiation hybrid mapping resources for non-mammalian vertebrates Nucleic Acids Res 1998 26 15 3562 3566 10.1093/nar/26.15.3562 9671819 PMC147736

[B45] Fukagawa T Hayward N Yang J Azzalin C Griffin D Stewart AF Brown W The chicken HPRT gene: a counter selectable marker for the DT40 cell line Nucleic Acids Res 1999 27 9 1966 1969 10.1093/nar/27.9.1966 10198428 PMC148408

[B46] Ekker M Speevak MD Martin CC Joly L Giroux G Chevrette M Stable transfer of zebrafish chromosome segments into mouse cells Genomics 1996 33 1 57 64 10.1006/geno.1996.0159 8617510

[B47] Karere GM Lyons LA Froenicke L Enhancing radiation hybrid mapping through whole genome amplification Hereditas 2010 147 2 103 112 10.1111/j.1601-5223.2010.02166.x 20536549 PMC4706074

[B48] Zhang L Cui X Schmitt K Hubert R Navidi W Arnheim N Whole genome amplification from a single cell: implications for genetic analysis Proc Natl Acad Sci USA 1992 89 13 5847 5851 10.1073/pnas.89.13.5847 1631067 PMC49394

[B49] Telenius H Carter NP Bebb CE Nordenskjold M Ponder BA Tunnacliffe A Degenerate oligonucleotide-primed PCR: general amplification of target DNA by a single degenerate primer Genomics 1992 13 3 718 725 10.1016/0888-7543(92)90147-K 1639399

[B50] Silander K Saarela J Whole genome amplification with Phi29 DNA polymerase to enable genetic or genomic analysis of samples of low DNA yield Methods Mol Biol 2008 439 1 18 10.1007/978-1-59745-188-8_1 18370092

[B51] Dean FB Hosono S Fang L Wu X Faruqi AF Bray-Ward P Sun Z Zong Q Du Y Du J Comprehensive human genome amplification using multiple displacement amplification Proc Natl Acad Sci USA 2002 99 8 5261 5266 10.1073/pnas.082089499 11959976 PMC122757

[B52] Lasken RS Genomic DNA amplification by the multiple displacement amplification (MDA) method Biochem Soc Trans 2009 37 Pt 2 450 453 19290880 10.1042/BST0370450

[B53] Qin J Jones RC Ramakrishnan R Studying copy number variations using a nanofluidic platform Nucleic Acids Res 2008 36 18 e116 10.1093/nar/gkn518 18710881 PMC2566873

[B54] Jang JS Simon VA Feddersen RM Rakhshan F Schultz DA Zschunke MA Lingle WL Kolbert CP Jen J Quantitative miRNA expression analysis using fluidigm microfluidics dynamic arrays BMC Genomics 2011 12 144 10.1186/1471-2164-12-144 21388556 PMC3062620

[B55] Mengual L Burset M Marin-Aguilera M Ribal MJ Alcaraz A Multiplex preamplification of specific cDNA targets prior to gene expression analysis by TaqMan Arrays BMC Res Notes 2008 1 21 10.1186/1756-0500-1-21 18710479 PMC2518557

[B56] Time Treehttp://www.timetree.org/index.php

[B57] Solinhac R Leroux S Galkina S Chazara O Feve K Vignoles F Morisson M Derjusheva S Bed'hom B Vignal A Integrative mapping analysis of chicken microchromosome 16 organization BMC Genomics 2011 11 616 21050458 10.1186/1471-2164-11-616 PMC3091757

[B58] Faraut T de Givry S Chabrier P Derrien T Galibert F Hitte C Schiex T A comparative genome approach to marker ordering Bioinformatics 2007 23 2 e50 56 10.1093/bioinformatics/btl321 17237105

[B59] Faraut T de Givry S Hitte C Lahbib-Mansais Y Morisson M Milan D Schiex T Servin B Vignal A Galibert F Contribution of radiation hybrids to genome mapping in domestic animals Cytogenet Genome Res 2009 126 1–2 21 33 20016154 10.1159/000245904

[B60] Hitte C Madeoy J Kirkness EF Priat C Lorentzen TD Senger F Thomas D Derrien T Ramirez C Scott C Facilitating genome navigation: survey sequencing and dense radiation-hybrid gene mapping Nat Rev Genet 2005 6 8 643 648 10.1038/nrg1658 16012527

[B61] Hitte C Kirkness EF Ostrander EA Galibert F Survey sequencing and radiation hybrid mapping to construct comparative maps Methods Mol Biol 2008 422 65 77 10.1007/978-1-59745-581-7_5 18629661 PMC2661178

[B62] Batellier F Marchal F Scheller MF Gautron J Sellier N Taouis M Monbrun C Vignal A Brillard JP Sex ratios in mule duck embryos at various stages of incubation Theriogenology 2004 61 2–3 573 580 14662153 10.1016/s0093-691x(03)00208-5

[B63] Echard GGJ Gillois M Localisation des gène MPI, PKM2,NP sur le chromosome3 de porc (Sus scrofa L.) et analyse cytogénétique d'une lignée de hamster chinois issuede la DON (Wg3h) Genet Sel Evol 1984 16 261 270 10.1186/1297-9686-16-3-261 22879165 PMC2714324

[B64] Primer3 http://frodo.wi.mit.edu/

[B65] UCSC In-Silico PCR http://genome.csdb.cn/cgi-bin/hgPcr

[B66] Servin B de Givry S Faraut T Statistical confidence measures for genome maps: application to the validation of genome assemblies Bioinformatics 2010 26 24 3035 3042 10.1093/bioinformatics/btq598 21076149

[B67] Voorrips RE MapChart: software for the graphical presentation of linkage maps and QTLs J Hered 2002 93 1 77 78 10.1093/jhered/93.1.77 12011185

[B68] Crooijmans RP Vrebalov J Dijkhof RJ van der Poel JJ Groenen MA Two-dimensional screening of the Wageningen chicken BAC library Mamm Genome 2000 11 5 360 363 10.1007/s003350010068 10790534

[B69] Yerle M Galman O Lahbib-Mansais Y Gellin J Localization of the pig luteinizing hormone/choriogonadotropin receptor gene (LHCGR) by radioactive and nonradioactive *in situ* hybridization Cytogenet Cell Genet 1992 59 48 51 10.1159/000133198 1733673

