# Supplementary material for: A duck RH panel and its potential for assisting NGS genome assembly
Source: BMC Genomics. 2012 Sep 28;13:513. doi: 10.1186/1471-2164-13-513 (PMC3496577; doi:10.1186/1471-2164-13-513)
Supplement: Additional File 2 — Figure S1. Checking the 13 largest scaffolds by RH mapping. Each thick horizontal line represents a scaffold; arrows point to the names of the markers which were genotyped on the duck RH panel. The approximate position of the markers is shown as well as the scaffold lengths. Markers in the same color and contained within the same box are linked by RH mapping. For the 12 first scaffolds shown, the RH mapping data confirm the scaffold assembly. The last one, scaffold504, was the only one which was detected to be discontinuous, as marker sca504F is not linked by RH mapping to the five other markers sca504A, sca504B, sca504C, sca504D and sca504E. Comparative analysis with chicken shows that the portion of the scaffold containing sca504F aligns to GGA2, whereas the rest aligns to GGA1. [file 1471-2164-13-513-S2.pdf]

13 Largest scaffolds > 4 Mb

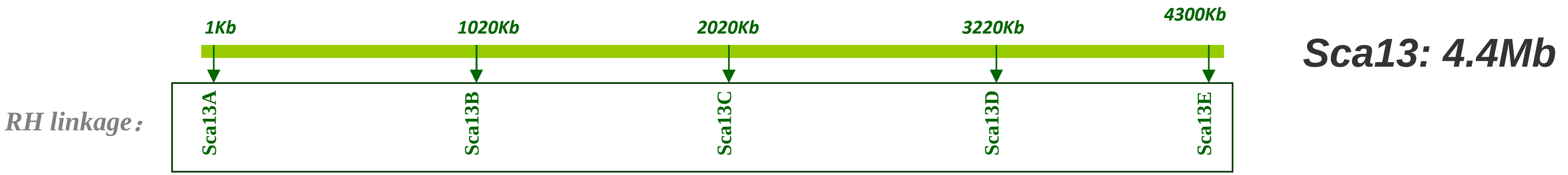

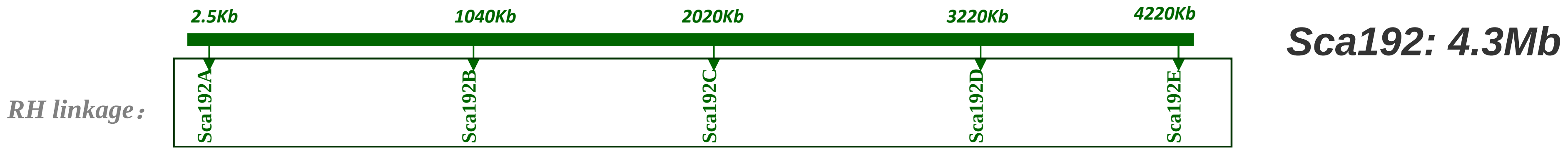

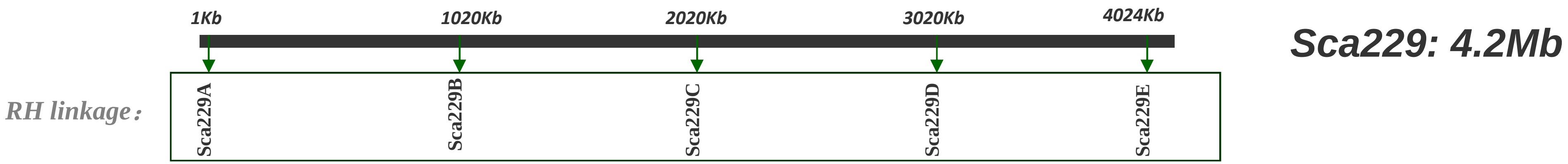

Markers scaXXX… contained in the same box are linked together by RH mapping.

13 Largest scaffolds > 4 Mb

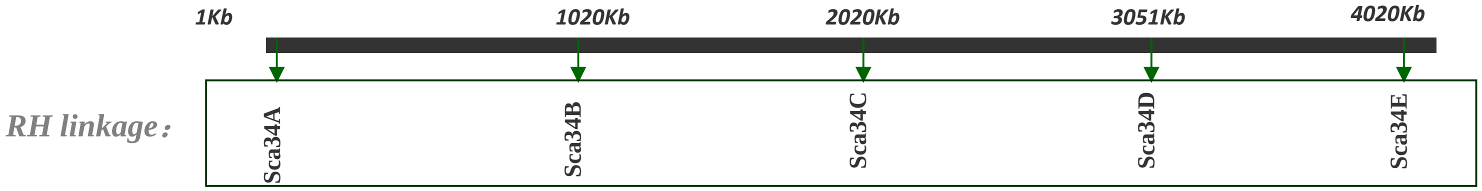

Sca34: 4.1Mb

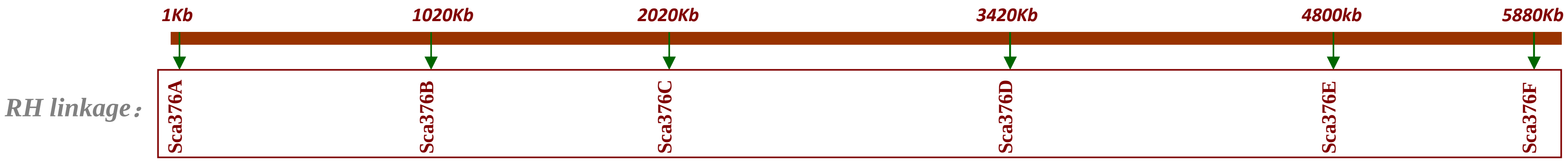

Sca376: 5.9Mb

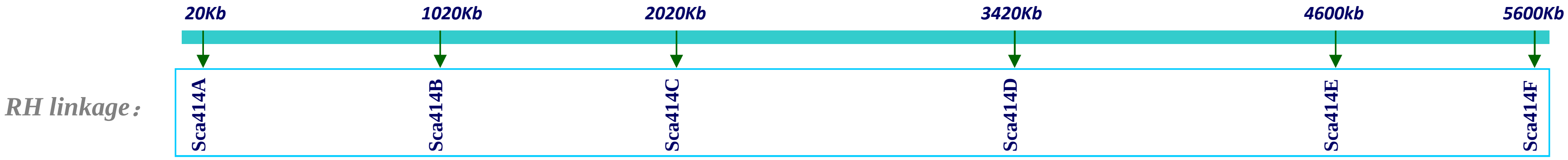

Sca414: 5.7Mb

13 Largest scaffolds > 4 Mb

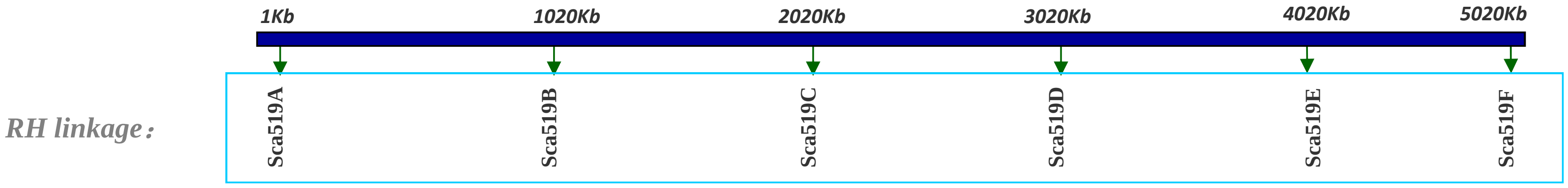

Sca519: 5Mb

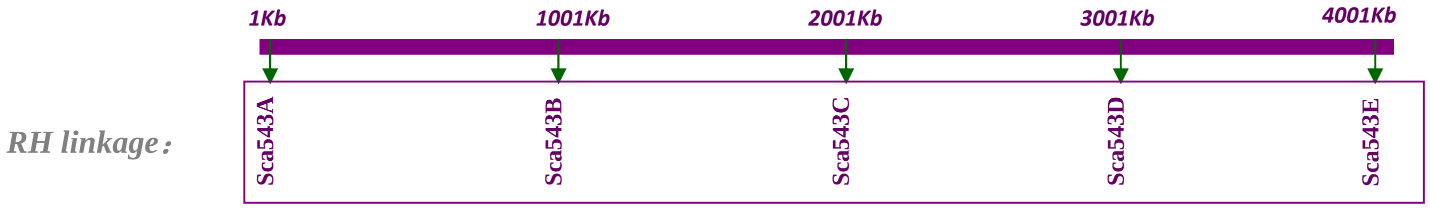

Sca543: 4Mb

13 Largest scaffolds > 4 Mb

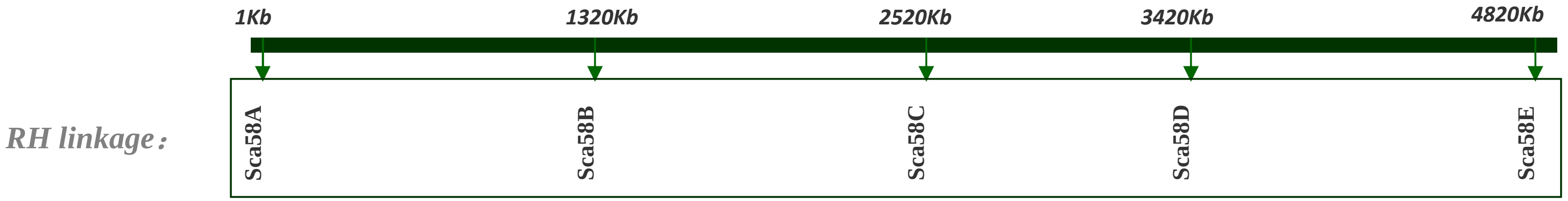

Sca58: 4.9Mb

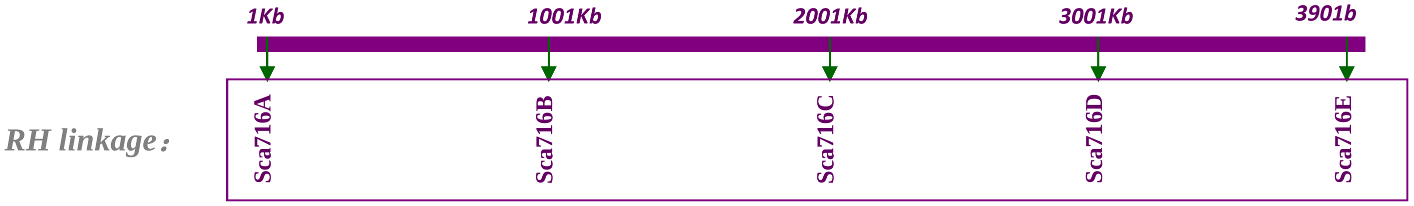

Sca716: 4Mb

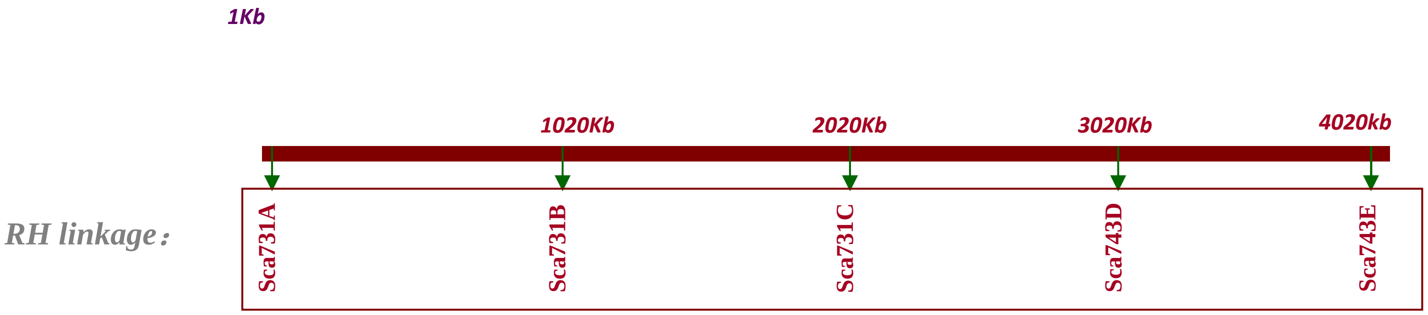

Sca731: 4.1Mb

13 Largest scaffolds > 4 Mb

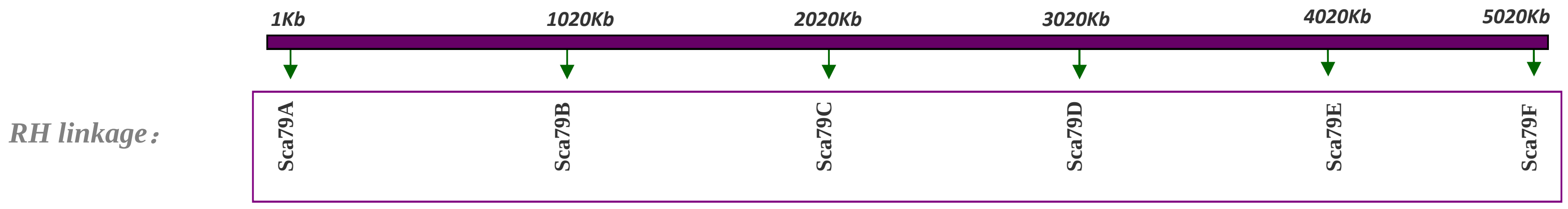

Sca79: 5.1Mb

13 Largest scaffolds > 4 Mb

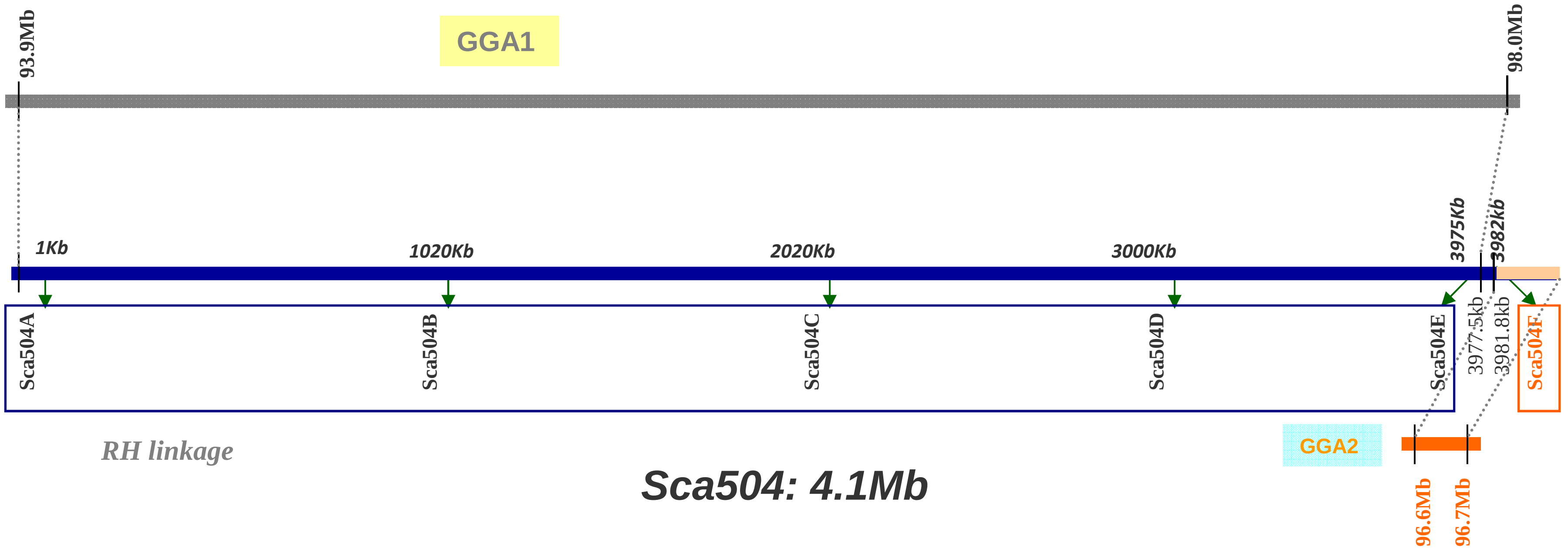

Markers sca504A, sca504B, sca504C, sca504D and sca504E are linked together by RH mapping, whereas marker sca504F is not linked, suggesting the end of the scaffold is misassembled.
