# Supplementary material for: A duck RH panel and its potential for assisting NGS genome assembly
Source: BMC Genomics. 2012 Sep 28;13:513. doi: 10.1186/1471-2164-13-513 (PMC3496577; doi:10.1186/1471-2164-13-513)
Supplement: Additional File 3 — Figure S2. Testing duck scaffolds aligning to two chicken chromosomes. Duck scaffolds are represented together with the portions of chicken chromosomes to which they show high sequence similarity in the Narcisse database. The approximate position of the markers on the scaffolds and on the chicken genome is shown as well as the scaffold lengths To test if the synteny breakpoints are due to an evolutionary chromosomal rearrangement or a problem in the assembly of scaffolds, a pair of markers was chosen close together on the scaffolds, but spanning the break points. Whenever markers are linked together by RH mapping, they are contained in the same box and are represented in the same colour. [file 1471-2164-13-513-S3.pdf]

# Duck scaffolds aligning to two chicken chromosomes

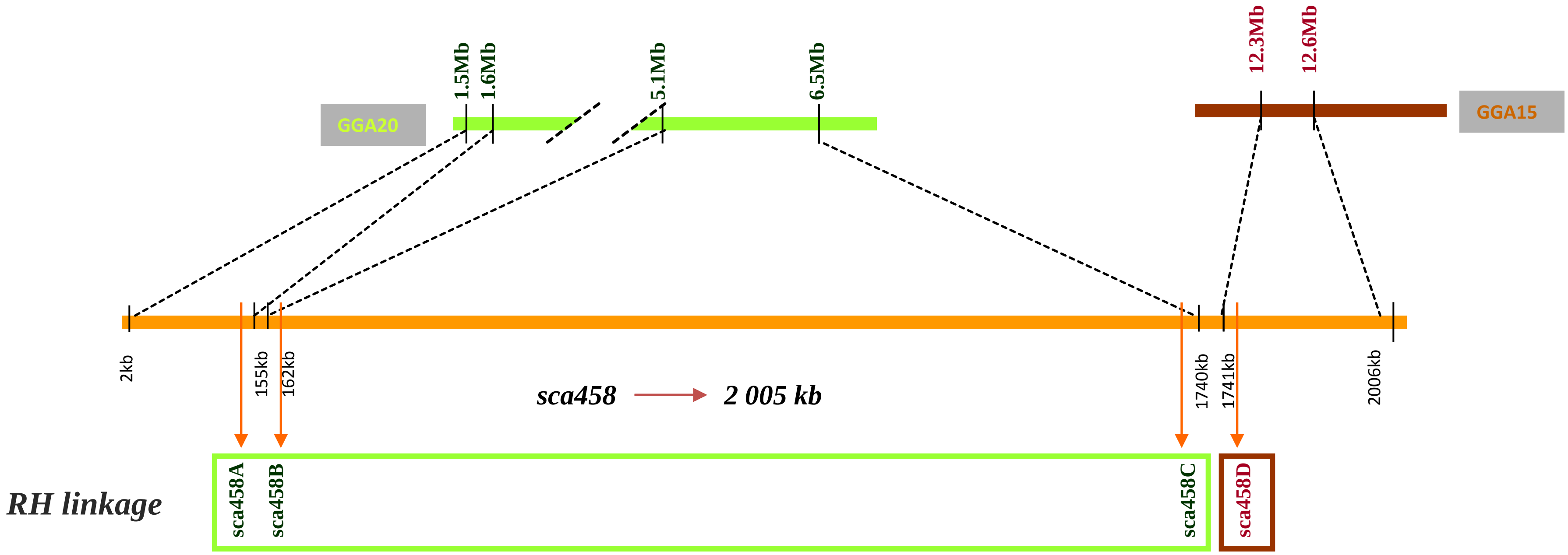


Markers scaXXX… contained in the same box are linked together by RH mapping.

# *Duck scaffolds aligning to two chicken chromosomes*

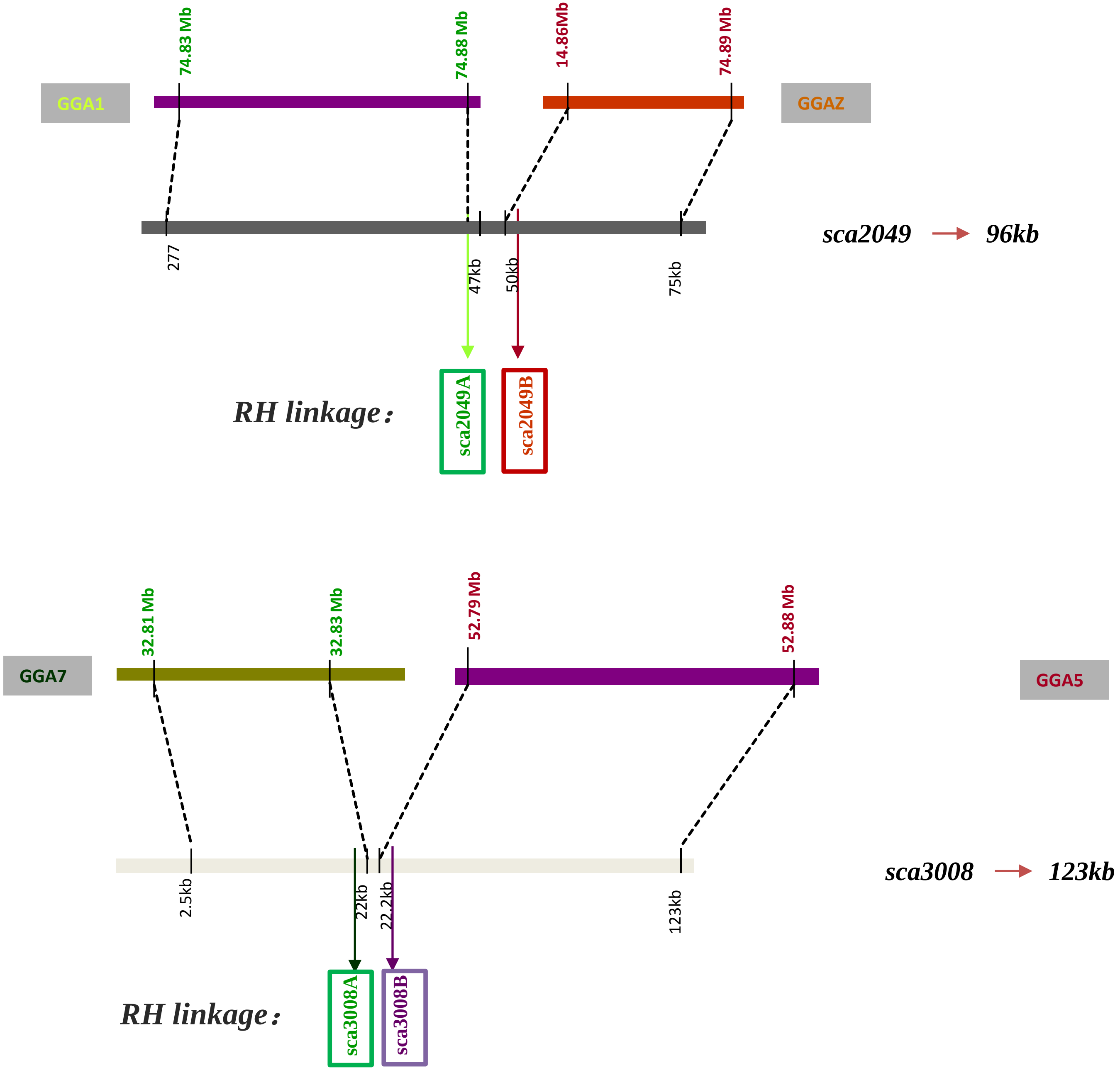

# Duck scaffolds aligning to two chicken chromosomes

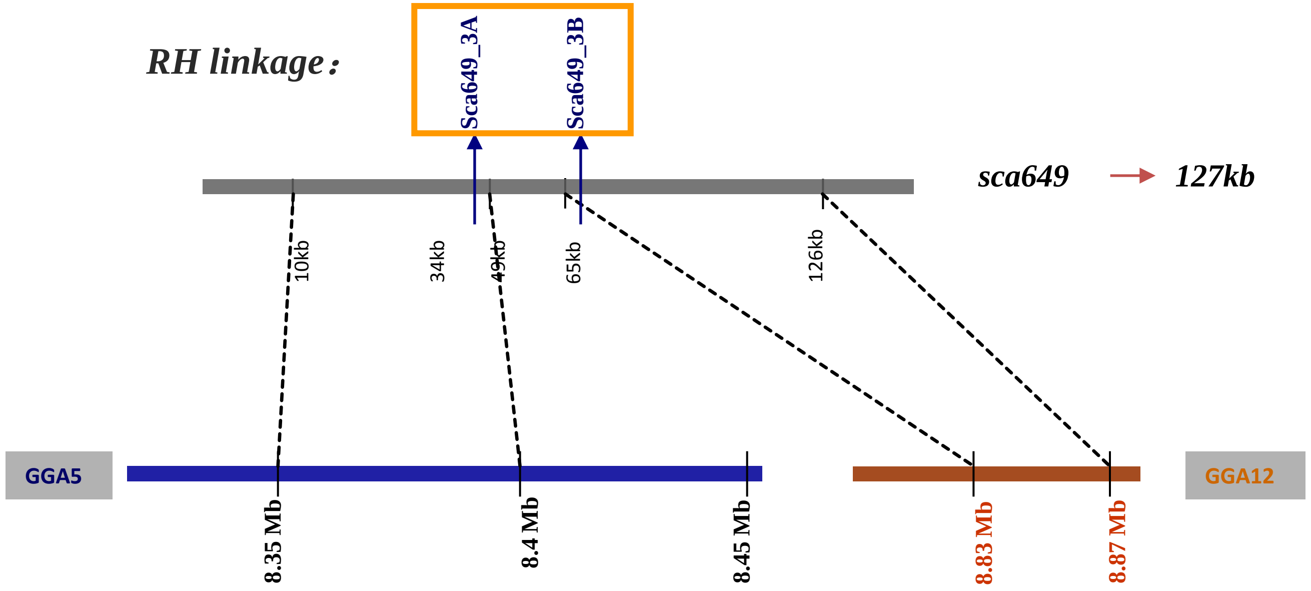

# Duck scaffolds aligning to two chicken chromosomes

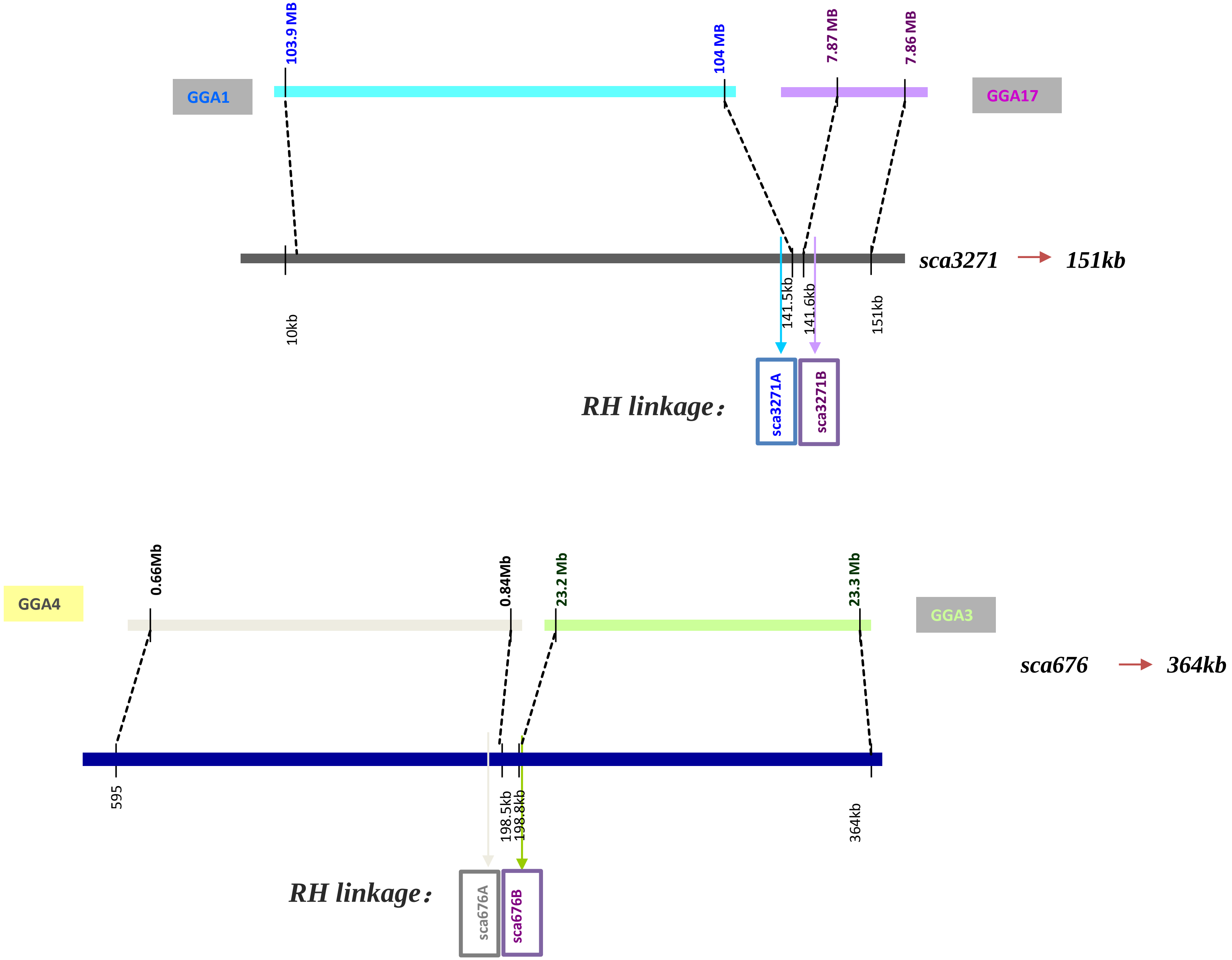

# Duck scaffolds aligning to two chicken chromosomes

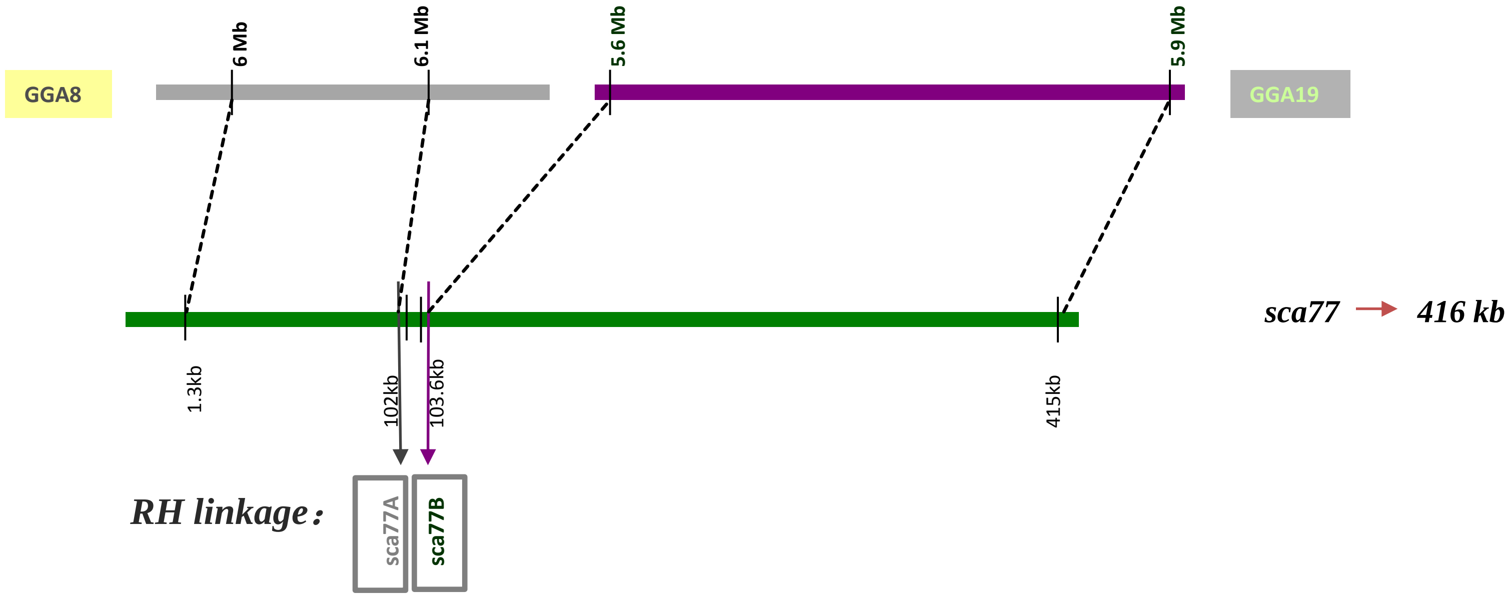


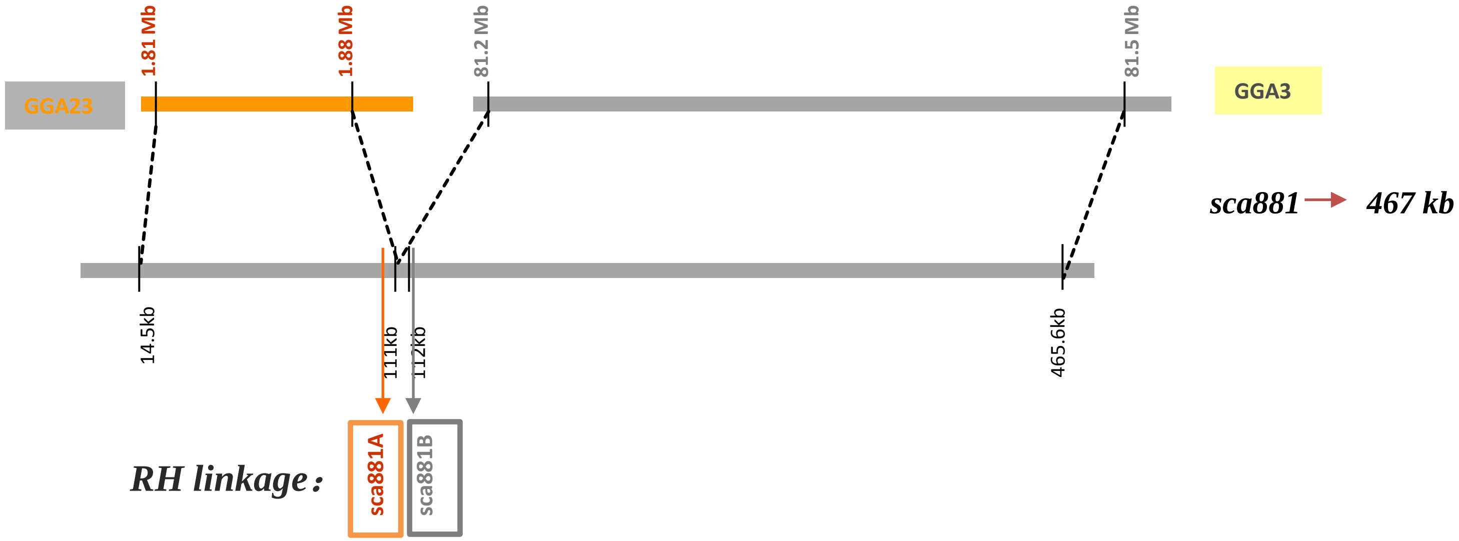

# Duck scaffolds aligning to two chicken chromosomes

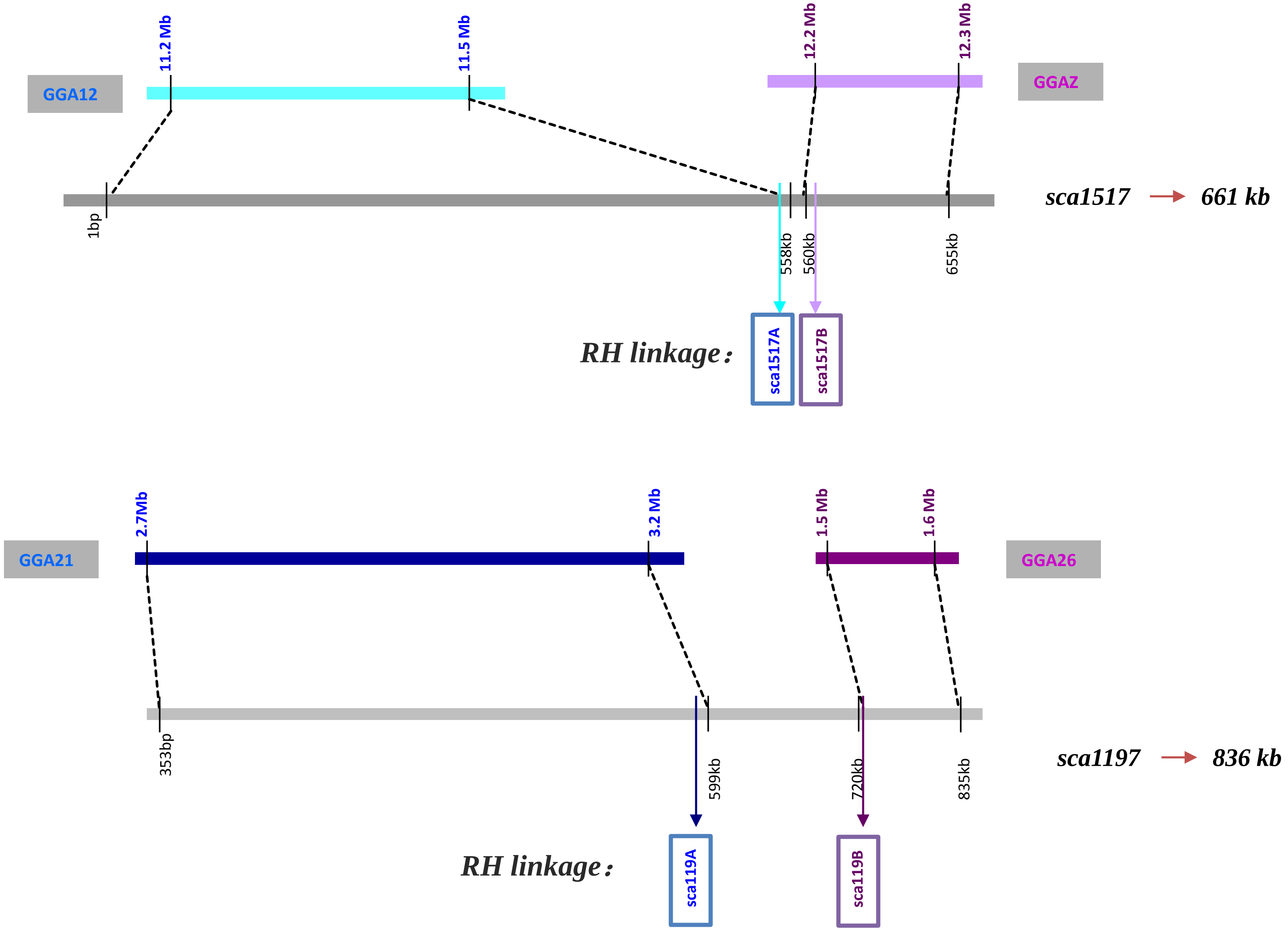

# Duck scaffolds aligning to two chicken chromosomes

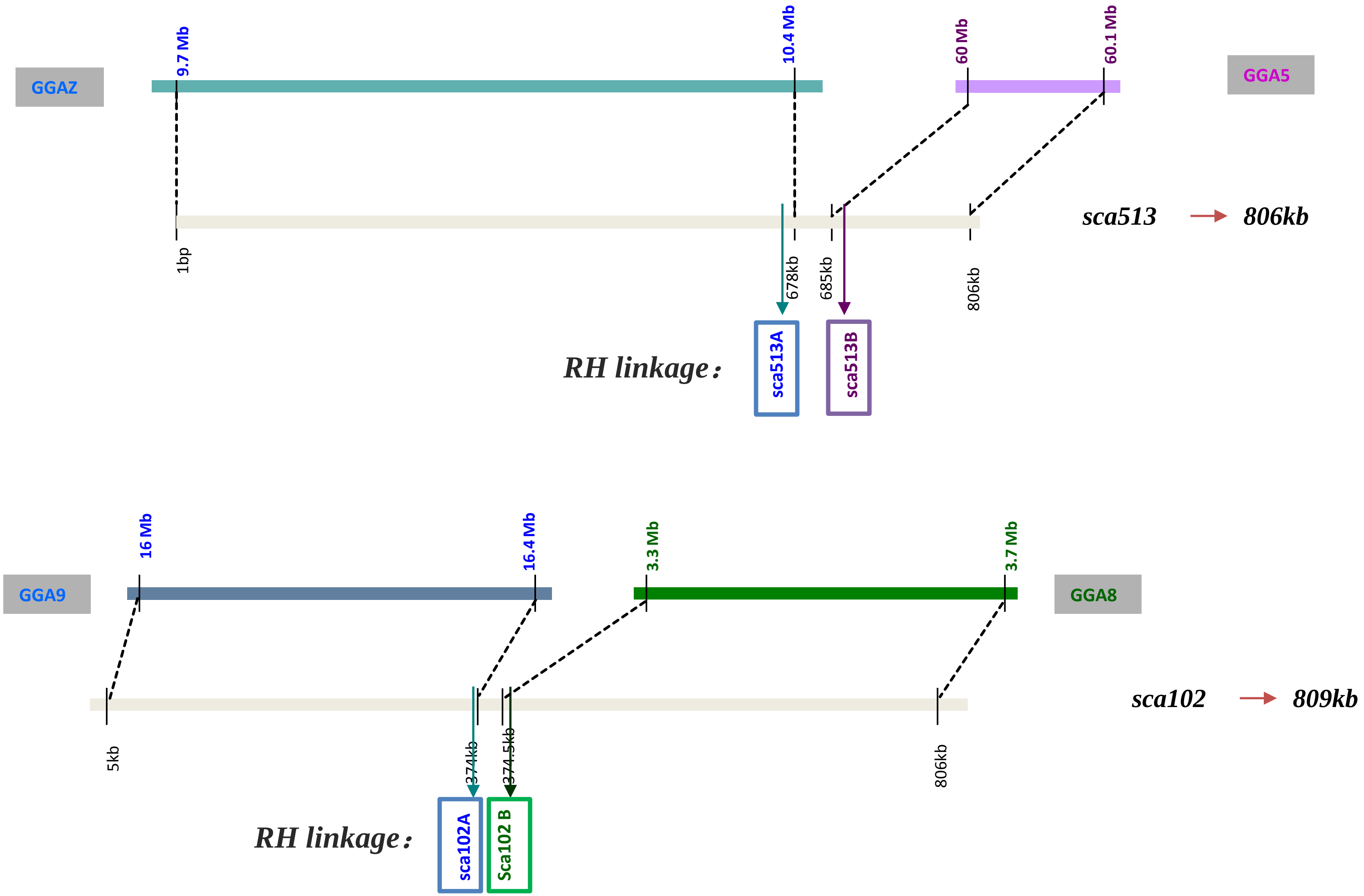

# Duck scaffolds aligning to two chicken chromosomes

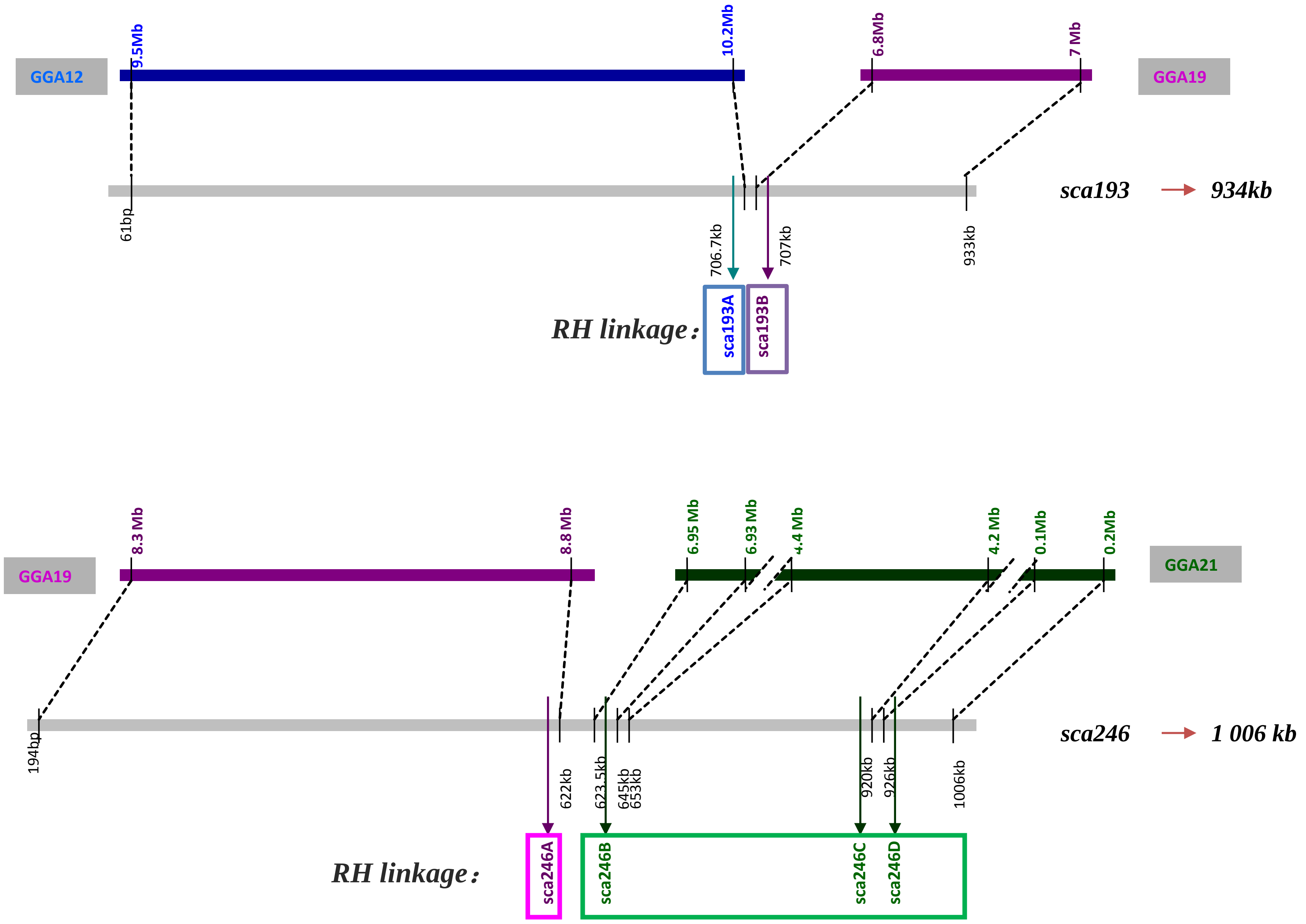

# *Duck scaffolds aligning to two chicken chromosomes*

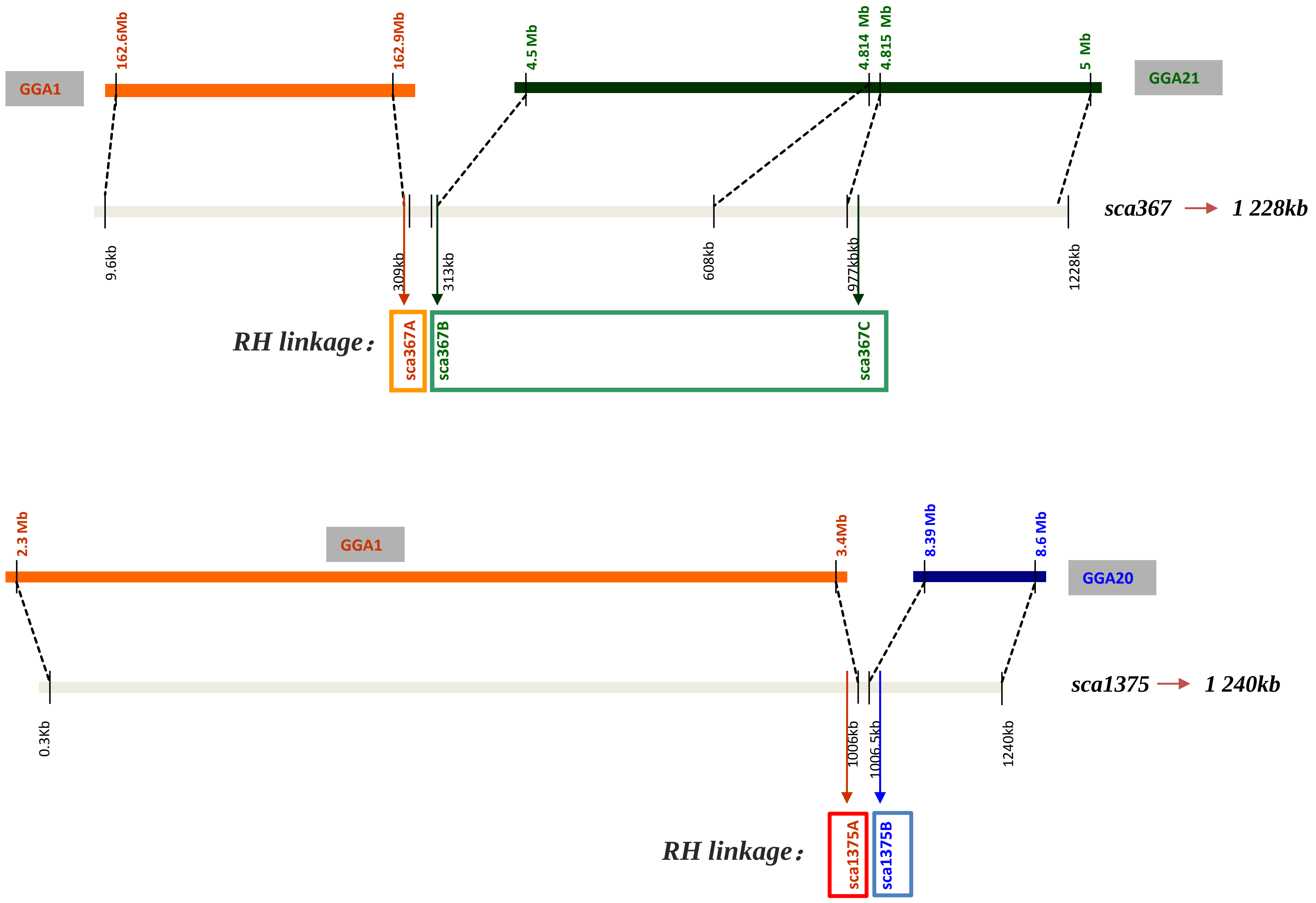

# *Duck scaffolds aligning to two chicken chromosomes*

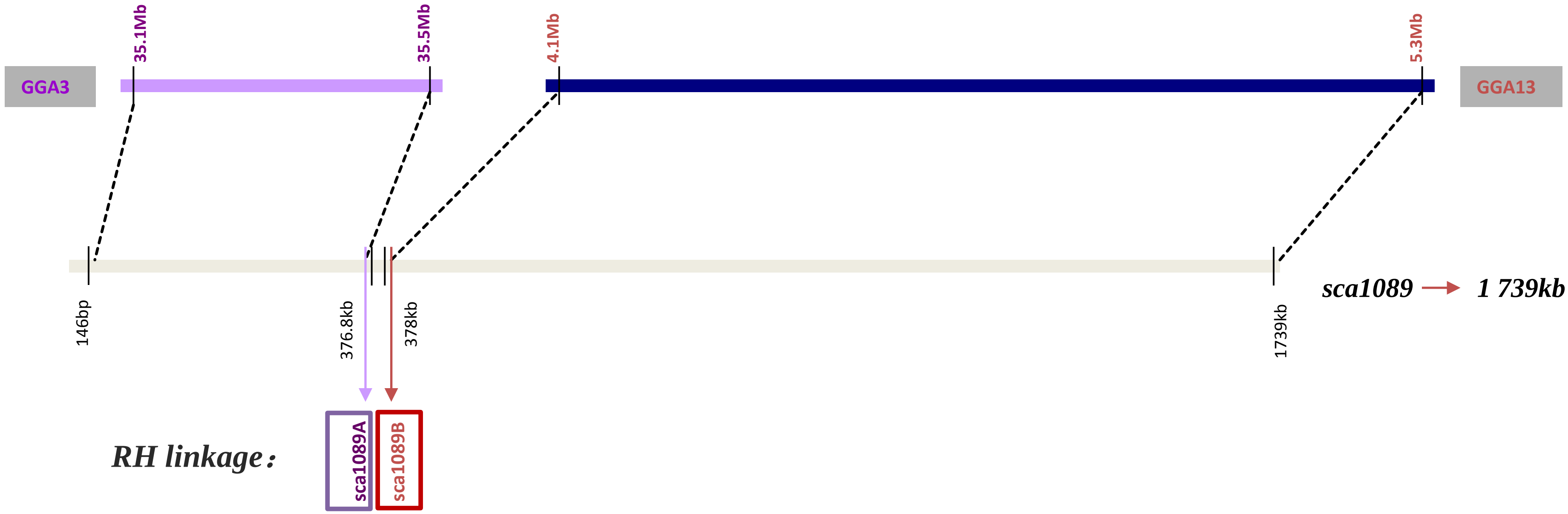

# Duck scaffolds aligning to two chicken chromosomes

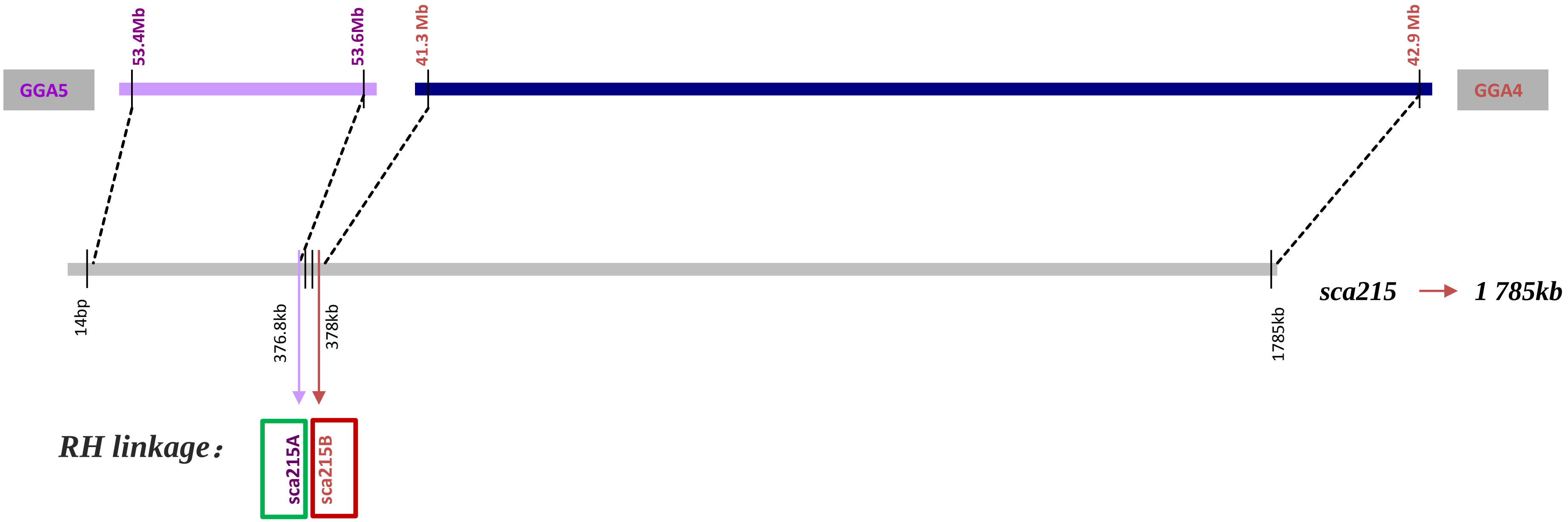

# *Duck scaffolds aligning to two chicken chromosomes*

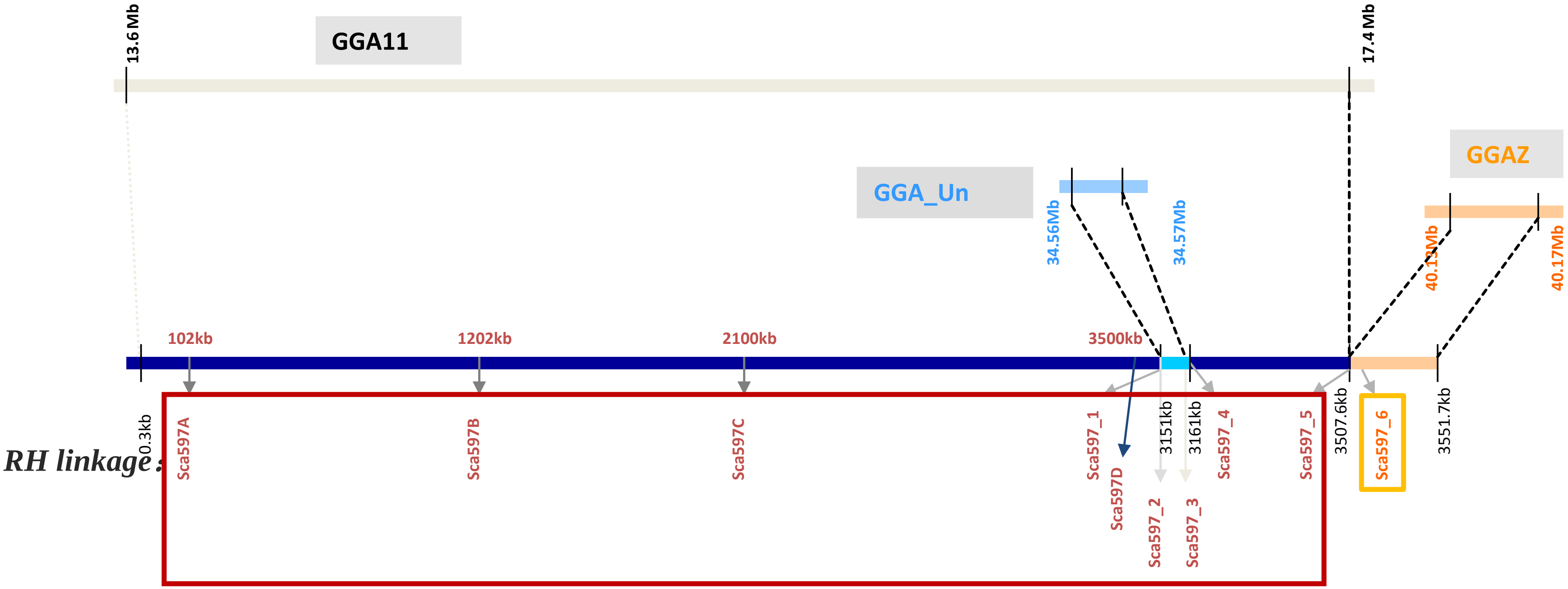


## *Sca597: 3.5Mb*

Scaffold597 was a large scaffold which was detected to be discontinuous as it could be mapped on different chicken chromosome as shown in the graph. The positions of all markers were also indicated. The coordinates on the breakpoints were given as well.
